# Lateralized Expression of Cortical Perineuronal Nets during Maternal Experience is Dependent on MECP2

**DOI:** 10.1523/ENEURO.0500-19.2020

**Published:** 2020-06-09

**Authors:** Billy Y. B. Lau, Dana E. Layo, Brett Emery, Matthew Everett, Anushree Kumar, Parker Stevenson, Kristopher G. Reynolds, Andrew Cherosky, Sarah-Anne H. Bowyer, Sarah Roth, Delaney G. Fisher, Rachel P. McCord, Keerthi Krishnan

**Affiliations:** Department of Biochemistry and Cellular and Molecular Biology, University of Tennessee, Knoxville, TN 37996

**Keywords:** alloparenting, lateralization, MECP2, perineuronal nets, Rett syndrome, somatosensory cortex

## Abstract

Cortical neuronal circuits along the sensorimotor pathways are shaped by experience during critical periods of heightened plasticity in early postnatal development. After closure of critical periods, measured histologically by the formation and maintenance of extracellular matrix structures called perineuronal nets (PNNs), the adult mouse brain exhibits restricted plasticity and maturity. Mature PNNs are typically considered to be stable structures that restrict synaptic plasticity on cortical parvalbumin+ (PV+) GABAergic neurons. Changes in environment (i.e., novel behavioral training) or social contexts (i.e., motherhood) are known to elicit synaptic plasticity in relevant neural circuitry. However, little is known about concomitant changes in the PNNs surrounding the cortical PV+ GABAergic neurons. Here, we show novel changes in PNN density in the primary somatosensory cortex (SS1) of adult female mice after maternal experience [called surrogate (Sur)], using systematic microscopy analysis of a whole brain region. On average, PNNs were increased in the right barrel field and decreased in the left forelimb regions. Individual mice had left hemisphere dominance in PNN density. Using adult female mice deficient in methyl-CpG-binding protein 2 (MECP2), an epigenetic regulator involved in regulating experience-dependent plasticity, we found that MECP2 is critical for this precise and dynamic expression of PNN. Adult naive *Mecp2*-heterozygous (Het) females had increased PNN density in specific subregions in both hemispheres before maternal experience, compared with wild-type (WT) littermate controls. The laterality in PNN expression seen in naive Het (NH) was lost after maternal experience in Sur Het (SH) mice, suggesting possible intact mechanisms for plasticity. Together, our results identify subregion and hemisphere-specific alterations in PNN expression in adult females, suggesting extracellular matrix plasticity as a possible neurobiological mechanism for adult behaviors in rodents.

## Significance Statement

Perineuronal nets (PNNs) are extracellular matrix structures that surround cortical parvalbumin+ (PV+) fast spiking GABAergic interneurons and synapses. They have long been considered stable structures that restrict synaptic plasticity. Removal of PNNs by enzymes reactivates plasticity in the rodent visual and auditory cortices and in the amygdala. However, it is currently unknown whether PNNs in adult brains undergo changes in expression under normal physiological conditions, similar to synaptic plasticity mechanisms. If they do, PNNs may not be very stable structures as they are perceived. We provide evidence that mature PNNs in the adult mouse primary somatosensory cortex (SS1) show dynamic expression changes in a hemisphere-specific, subregion-specific manner after maternal experience and are regulated by methyl-CpG-binding protein 2 (MECP2).

## Introduction

Perineuronal nets (PNNs) are specialized extracellular matrix structures that can act as physical barriers or modulators of plasticity, restrict axon regeneration, and form molecular brakes that actively control synaptic maturation and the function of cortical parvalbumin+ (PV+) GABAergic interneurons that drive γ oscillations (Nakagawa et al., 1986; Kosaka and Heizmann, 1989; Hartig et al., 1992; Bartos et al., 2002; Deepa et al., 2002; Pizzorusso et al., 2002, 2006; Dityatev et al., 2007; Frischknecht et al., 2009; Sugiyama et al., 2009; Gundelfinger et al., 2010; Durand et al., 2012; Orlando et al., 2012; Suttkus et al., 2012; Donato et al., 2013; Vo et al., 2013; Ye and Miao, 2013; Krishnan et al., 2015, 2017; Bernard and Prochiantz, 2016; Carstens et al., 2016; de Winter et al., 2016; Begum and Sng, 2017; Hou et al., 2017; Kalemaki et al., 2018; Ueno et al., 2018; Sigal et al., 2019). In rodents, mature PNNs in the adult cortex are thought to be stable structures, inhibitory to plasticity, and perhaps play roles in long-term memory such as “engrams” (Gogolla et al., 2009; Carstens et al., 2016; Thompson et al., 2018). However, most of these observations are based on postnatal cortical development (when typical connections in neural circuitry are still forming) and models for neurobiological disorders (where neural circuitry development and function have gone awry). Currently, it is unclear whether changes in adult PNN expression occur under normal conditions and behavioral contexts.

PNNs are composed of chondroitin sulfate proteoglycans, hyaluronan glycosaminoglycan chains, link proteins and tenascin-R (Bignami et al., 1992; Carulli et al., 2007; Kwok et al., 2010; Miyata and Kitagawa, 2017). Wisteria floribunda agglutinin (WFA) is commonly used as a marker for PNNs in the cortex and other brain regions (Hartig et al., 1992; Brückner et al., 1996). WFA specifically binds to *N*-acetyl galactosamine found on most chondroitin sulfate side chains of chondroitin sulfate proteoglycans. In rodents, WFA-labeled PNNs are localized predominantly around soma and proximal dendrites of PV+ GABAergic interneurons of the mature cortex. They interdigitate with synaptic contacts on cortical PV+ GABAergic neurons and regulate experience-dependent synaptic plasticity in the cortex, hippocampus, and amygdala (Gogolla et al., 2009; Miyata et al., 2012; Krishnan et al., 2015, 2017; Cattaud et al., 2018; Murthy et al., 2019; Sigal et al., 2019).

In the human brain, decreased numbers of PNNs are associated with pathologic conditions such as decreased memory and motor agility (Morawski et al., 2004; Brückner et al., 2008; Cabungcal et al., 2013; Pantazopoulos et al., 2015; Enwright et al., 2016). Mouse models for varying neurologic disorders show abnormal/atypical expression of PNNs which, when removed, can greatly improve the associated pathology or behavioral readouts in these models (Berretta et al., 2015; Pizzo et al., 2016; Krishnan et al., 2017; Reinhard et al., 2019). We have previously shown that precocious or atypical expression of PNNs caused sensory processing deficits in developing male or adult female mouse models for Rett syndrome, respectively (Krishnan et al., 2015, 2017). Rett syndrome is a neuropsychiatric disorder predominantly caused by mutations in the X-linked gene, methyl-CpG-binding protein 2 (MECP2; Amir et al., 1999). MECP2 regulates neuronal chromatin architecture and gene transcription in response to neural activity and experience during postnatal life (Zhou et al., 2006; Chahrour et al., 2008; Skene et al., 2010; Ebert et al., 2013; Becker et al., 2016). The known cellular function of MECP2 and the characteristic timing of disease progression led us to hypothesize that MECP2 regulates experience-dependent plasticity in specific neural circuits during windows of enhanced sensory and social experience throughout life; disruptions in timing of these plasticity mechanisms results in atypical responses in behavior. We previously tested this hypothesis using a pup retrieval task in the alloparental care paradigm (Krishnan et al., 2017).

Parenting is an ethologically relevant social behavior consisting of stereotypic components involving the care and nourishment of young. First-time dams seek and gather wandering/scattered pups back to the nest (pup retrieval), an essential aspect of maternal care. Pup retrieval involves processing of primary sensory cues (auditory, tactile, olfactory) to direct efficient searching and gathering of pups with goal-directed movements back to the nest (Beach and Jaynes, 1956; Stern, 1996; Lonstein et al., 2015). Virgin female mice (naive) with no previous maternal experience can execute efficient pup retrieval after co-housing [surrogates (Sur)] with a first-time mother and her pups (Cohen et al., 2011a). This assay allows for interrogation of adult experience-dependent plasticity mechanisms as well as likely non-hormonal, epigenetic mechanisms involving the sensory and motor neural circuits (Stolzenberg and Champagne, 2016). The role of the auditory cortex in pup retrieval and maternal experience is a topic of investigation in many labs (Stiebler et al., 1997; Galindo-Leon et al., 2009; Cohen et al., 2011a; Marlin et al., 2015). They continue to contribute to the understanding of how relevant sensory cues are processed in the maternal brain.

Previously, we found that atypical increase in PNN expression in the auditory cortex of *Mecp2*-heterozygous (Het) females caused inefficient pup retrieval (Krishnan et al., 2017). However, we did not find discernable changes in PNN density in the wild-type (WT) auditory cortex after successful completion of the pup retrieval task. On the one hand, as PNNs are considered barriers to plasticity, we anticipated reduction in PNN expression in WT that could facilitate efficient retrieval. On the other hand, there are no known reports of reduction in PNN expression in normal adult WT brains. Here, we seek to answer whether mature PNNs are maintained as stable structures or undergo dynamic expression changes in the primary somatosensory cortex (SS1) of adult female mice after maternal experience. We focused on the SS1 due to its known roles in tactile sensation, which is also important for efficient pup retrieval (Kenyon et al., 1981; Morgan et al., 1992; Feldman and Brecht, 2005; Brecht, 2007). By using WFA as a marker and whole-brain analysis of SS1, we find that mature PNNs in adult SS1 (1) are differentially expressed in a hemisphere-specific and subregion-specific manner; (2) show dynamic expression changes after maternal experience; and (3) are influenced by MECP2, a DNA methylation reader/epigenetic regulator of chromatin and gene expression.

## Materials and Methods

### Animals

All experiments were performed in adult female mice (10–12 weeks old) that were maintained on a 12/12 h light/dark cycle (lights on 07 A.M.) and received food *ad libitum*. Genotypes used were CBA/CaJ, *Mecp2^Het^* (C57BL/6 background; B6.129P2(C)-*Mecp2^tm1.1Bird^*/J) and *Mecp2^WT^*-siblings (Guy et al., 2001). All procedures were conducted in accordance with the National Institutes of Health’s Guide for the Care and Use of Laboratory Animals and approved by the University of Tennessee-Knoxville Institutional Animal Care and Use Committee.

### Pup retrieval behavior

**Figure 1.**
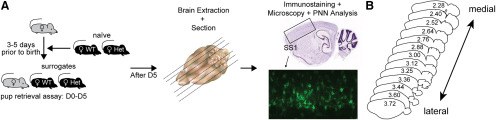
Schema representing behavioral and histology pipeline. (***A***) (Left) Alloparental behavioral model in mice. Pregnant CBA/CaJ female (grey mouse) is cohoused with adult female naïve WT and naïve Het littermate controls, which changes their status to surrogates, 3-5 days before birth of pups. Once pups are born, pup retrieval assay is performed with the surrogates from day 0 (D0) to 5 (D5). (Center) After behavioral experiments on D5, surrogate mice and age-matched naïve counterparts are perfused, their brains extracted, and sectioned as a single cohort. (Right) Standard immunostaining and imaging with epifluorescent slide scanner are performed, to image and analyze PNNs, as elaborated in Methods. (***B***) Schema of mouse brain sections cut in sagittal orientation, depicting all medial (2.28 mm, corresponding to map number 120) to lateral (3.72 mm, corresponding to map number 132) regions of SS1 analyzed for this study. Coordinate maps are based on Paxinos and Franklin’s mouse brain atlas, 4^th^ edition.

Pup retrieval behavior was performed as previously described (Krishnan et al., 2017; Fig. 1*A*). Briefly, we housed two virgin female mice (one *Mecp2^WT^* and one *Mecp2^Het^* termed “naive,” NW and NH, respectively) with a first-time pregnant CBA/CaJ female beginning 1–5 d before birth. Upon cohousing, the two naive mice are now termed Sur (SW for Sur *Mecp2^WT^* and SH for Sur *Mecp2^Het^*). Pup retrieval behavior started on the day the pups were born (postnatal day 0; D0) as follows for each adult Sur mouse: (1) habituation phase, one adult mouse was habituated with three to five pups in the home cage for 5 min; (2) isolation phase, pups were removed from the cage for 2 min; and (3) retrieval phase, pups were returned to home cage, one placed at each corner and at the center (the nest was left empty if there were fewer than five pups). Each adult female had maximum of 10 min to gather the pups to the original nest. After testing, all animals and pups were returned to the home cage. The same procedure was performed again daily to day 5. All behaviors were performed in the dark, during the light cycle (between 9 A.M. and 6 P.M.) and were video recorded.

### Immunohistochemistry

Immediately after the behavioral trial on day 5, Sur mice as well as a set of corresponding naive *Mecp2^WT^* and *Mecp2^Het^* mice were perfused with 4% paraformaldehyde/PBS, and brains were extracted and postfixed overnight at 4°C. Brains were then treated with 30% sucrose/PBS overnight at room temperature (RT) and sectioned in sagittal orientation using a freezing microtome at 70 μm. Free-floating brain sections were immunostained at RT as previously described in Krishnan et al. (2017), with a few modifications. Briefly, sections were blocked in 10% normal goat serum and 0.5% Triton X-100 for 3 h, then incubated with biotin-conjugated WFA Lectin (labels PNNs; 1:500; Sigma-Aldrich) and rabbit MECP2 primary antibody (1:1000; Cell Signaling) overnight at RT in a 5% normal goat serum and 0.25% Triton X-100 solution. Then, sections were incubated for 4 h with Alexa Fluor 488 and Texas-Red secondary antibodies (1:1000; Invitrogen) in a 5% normal goat serum and 0.25% Triton X-100 solution. Finally, sections were counterstained with the nuclear marker, DAPI (1:1000) for 5 min, and mounted in Fluoromount-G (Southern Biotech).

### Image acquisition and analysis

To analyze PNNs, 10× single-plane PNN images of the entire SS1 from each brain slice were acquired on a motorized stage, epifluorescent microscope (Keyence BZ-X710; Keyence Corp.) and stitched using BZ-X Analyzer (Keyence; Fig. 1*B*). Our initial observation of PNN intensity showed that SH had the most intense fluorescent signal in SS1. Thus, imaging settings were established based on SH within each cohort of mice, to minimize overexposure. The light exposure time for fluorescent signal acquisition was identified by finding the exposure time where a saturated pixel first appears within frame, then decreasing the exposure time by 1 unit, according to software specifications. This exposure time determination method was applied to all brain sections of SH in each cohort and the mean exposure time was calculated and used for final image acquisition within each cohort.

For PNN image analysis, each stitched image was opened in ImageJ (Schneider et al., 2012). Then, SS1 and somatosensory subregion areas were (1) mapped by overlaying templates from Paxinos and Franklin’s *The Mouse Brain*, fourth edition (Paxinos and Franklin, 2013), (2) outlined, and (3) measured using the functions in ImageJ. To count high-intensity mature PNNs, the Contrast setting from the browser was set to the far right to threshold weaker signals. The remaining signals were manually quantified under the classification that a “mature” PNN is at least 80% of its original shape (before contrast adjustment). Detailed protocol for mapping and counting procedures are now available at dx.doi.org/10.17504/protocols.io.bcf8itrw. All statistical analyses and graphs were generated using GraphPad Prism.

For MECP2 expression analysis, a portion of SS1 region was imaged using 20× objective and Keyence microscope, with tiled and stacks of 11 images, which were then collapsed and stitched using the Keyence stitch function. Imaging settings were based on left hemisphere of one brain and applied to the right hemisphere of the same brain. Measurement of MECP2 intensity was perform using ImageJ as follows: (1) for each tiled projection image, a region of interest (ROI) box of 1.5 mm (rostral-caudal) × 0.5 mm (dorsal-ventral) to select a large portion of SS1, which covers Layers II/III–V where most of the PNNs are localized; (2) individual intensity values were acquired and saved by selecting Analyze, Tools and Save XY Coordinates; and (3) exported Excel values were sorted and percentage cumulative frequency distribution were calculated using Microsoft Excel and GraphPad Prism (version 8).

### Principal component analysis (PCA) on PNN density

We used PNN densities (counts per area) for the entire SS1 across all individuals in the five cohorts. If multiple sections per map number were present, values were averaged across sections to give a single density. In the first PCA, to determine whether the PNN patterns segregated primarily by cohort or (genotype and experience), we kept the data for each individual animal separate and averaged PNN densities across every set of two adjacent map regions. Because the scale of raw numbers varied so widely between individual animals, we next median normalized PNN densities within each animal. To do this, we calculate the median PNN density across all map numbers for each individual. Then, each individual PNN density for each map number of that individual was divided by (“normalized by”) this median. Finally, these median normalized densities were averaged across all individuals in the same condition. This method of normalization accounts for the variability in the median across cohorts within the same condition. Some regions for some individuals had no data, and, because PCA does not tolerate missing data, we imputed these missing values by using the average median normalized values for that region from animals in other cohorts in the same condition. By running PCA in R, we obtained a weight for each map number showing the highest variance patterns in PNN densities across the individual animals. We then calculated the projection of each animal onto principle components 1 and 2. K-means clustering was used to calculate the two most evident clusters in this PCA space.

In the second PCA, to determine the major PNN density patterns that distinguish genotypes and experience conditions, we left data for each map number separate, but averaged the median normalized PNN densities across the five cohorts for each condition. We then performed PCA to determine the major patterns that distinguish these conditions.

## Results

### PNN density changes across somatosensory cortical maps

As somatosensation during pup retrieval primarily involves facial/snout areas in Sur (non-lactating adult females; Morgan et al., 1992; Stern, 1996; Lonstein and Stern, 1997), we present data collected from the somatosensory cortical regions involved in processing tactile stimuli in the alloparental care paradigm (Fig. 1*A*). According to Paxinos and Franklin’s atlas (fourth edition), there are eight different anatomic somatosensory cortical subregions [S1; S1 barrel field (S1BF), S1 dysgranular zone (S1DZ), S1 fore limb (S1FL), S1 jaw (S1J), S1 upper lip (S1ULp), S1 trunk (S1Tr), S1 hindlimb (S1HL) and undefined somatosensory cortex S1], here collectively called SS1 (Paxinos and Franklin, 2013). To determine how PNN expression changes across the different SS1 subregions, we took a systematic approach covering the whole SS1, rather than the standard approach of analyzing “representative sample sections” (Fig. 1*B*). We analyzed 40–60 sagittal brain sections (at 70 μm each, both left and right hemispheres) per animal, in five biological replicates across four conditions [naive WT (NW), naive Het (NH), Sur WT (SW), Sur Het (SH); Fig. 1*A*], as qualitative regional differences in PNN density were observed in pilot studies in our lab. The SS1 subregions are represented by map numbers 120–132, corresponding to lateral coordinates 2.28 mm (medial region) to 3.72 mm (lateral region), which encompass ∼1.5 mm of one mouse brain hemisphere.

**Figure 2.**
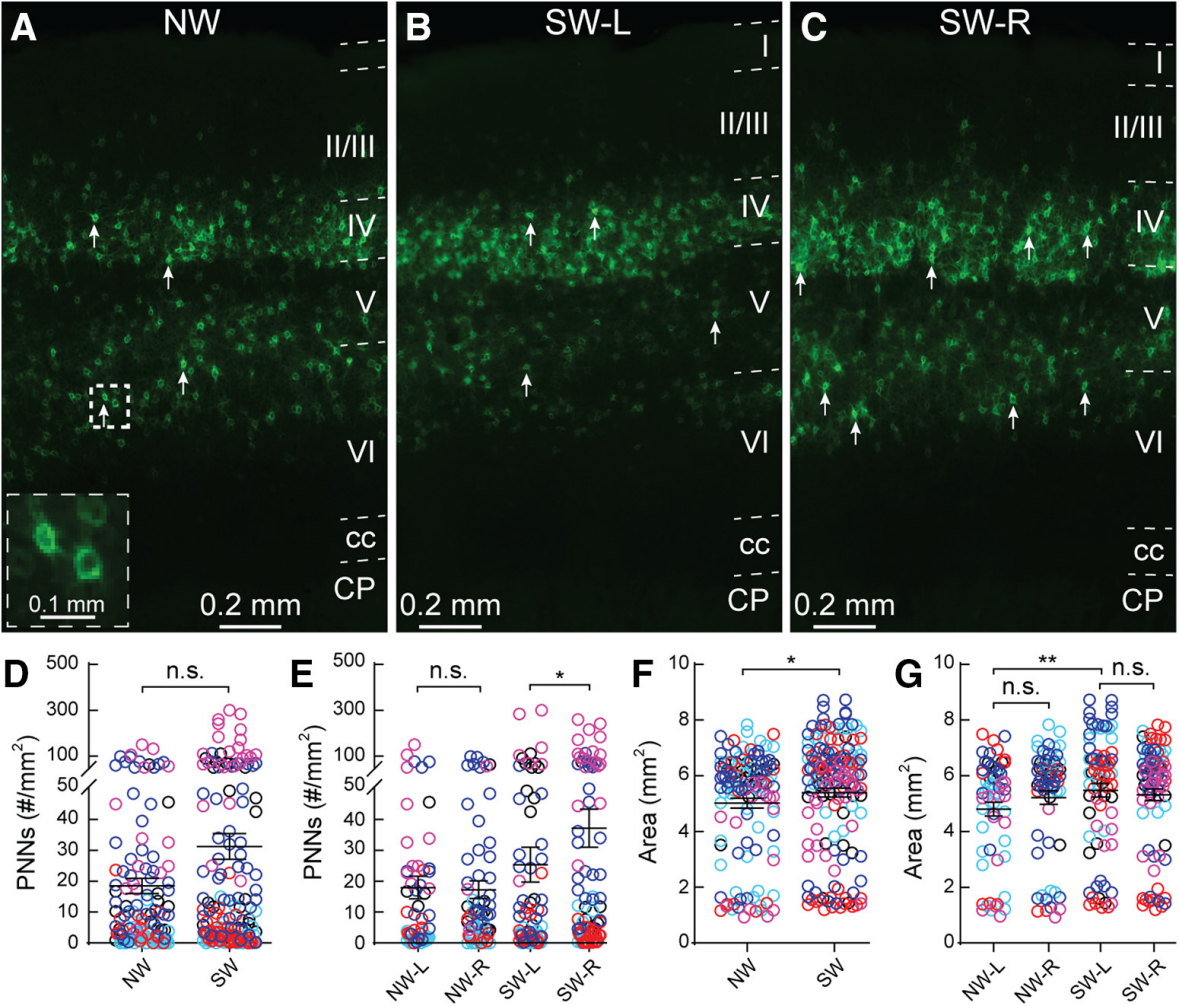
Surrogate *Mecp2^WT^* (*Wild type*) mice exhibit hemisphere specific increase in PNN density in the primary somatosensory cortex (SS1). (***A-C***) Representative epifluorescent images of PNN expression in SS1 of naïve WT (NW) (***A***) as well as left (L) (***B***) and right (R) (***C***) hemispheres of surrogate WT (SW). Layers 1 through 6 are outlined. CP = caudate putamen and cc = corpus callosum. Arrows indicate examples of high-intensity PNNs analyzed for the study. Inset in A shows magnified PNN structures in the box. (***D***) Combined hemisphere analysis of the density of high-intensity PNNs was not significantly different between NW and SW (NW: *n* = 123 images; SW: *n* = 162; *Mann-Whitney test*, *p* > 0.05). (***E***) Separate analysis of left and right hemisphere revealed a significant increase of high-intensity PNNs in the right hemisphere of SW (SW-R; *n* = 81 images) compared to the left hemisphere of SW (SW-L; *n* = 81 images) (*Kruskal-Wallis followed by Dunn’s test*, *p < 0.05), while no significant difference was observed between hemispheres of NW (NW-L: n = 59 images; NW-R: *n* = 64 images; Mann-Whitney test, *p* > 0.05). (***F,G***) Size of SS1 was overall significantly larger in SW compared to NW (F) (NW: *n* = 123 images; SW: n = 162 images; *Mann-Whitney test*, *p < 0.05). Enlargement of SS1 in SW occurred in the left hemisphere (G) (NW-L: *n* = 59 images, NW-R: *n* = 64 images; SW-L: *n* = 81 images; SW-R: *n* = 81 images; *Kruskal-Wallis* *followed by Dunn’s test*, **p < 0.01). For D-G, n.s. = not significant. Different colors represent each of the five cohorts. Each open circle represents PNN density in an individual brain section.

**Figure 3.**
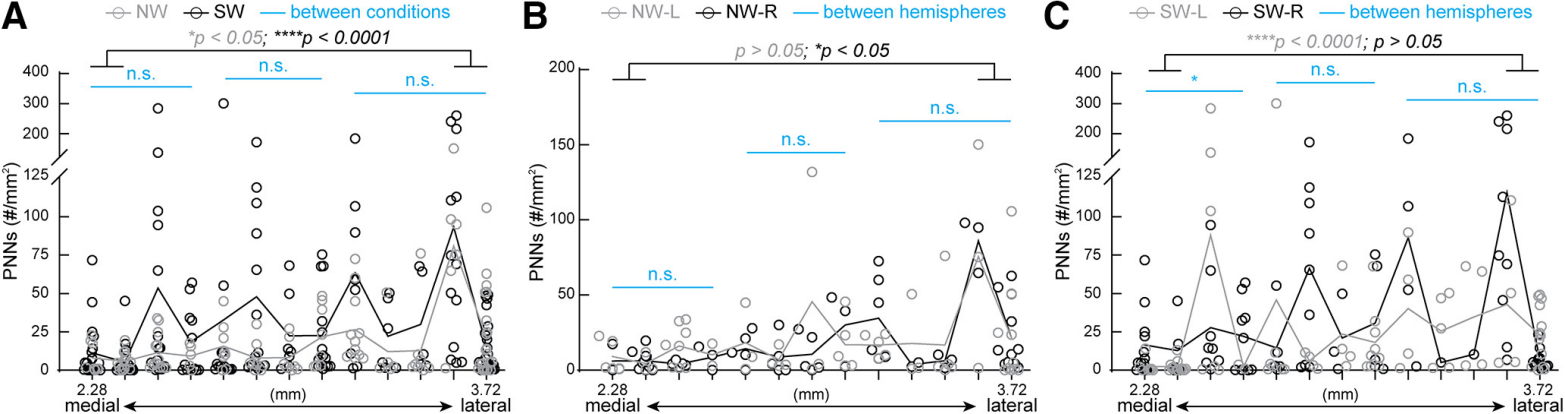
Dynamic changes in PNN density across medial-lateral axis of both naïve and surrogate WT SS1. (***A***) Distribution of combined PNN density from left and right hemispheres of SS1 revealed an increase of PNN density in lateral sections compared to the medial sections of the same condition. Statistical analysis revealed significant differences between the combined two most medial regions and the combined two most lateral regions within conditions (grey *p* value denotes NW: *n* = 22 images for medial, 28 images for lateral; black *p* value denotes SW: *n* = 37 images for medial, 37 images for lateral). This medial-lateral comparison analysis method also applies to all subsequent figure panels. No significant difference in PNN density was found between conditions, represented by the light blue lines. Statistical analysis of the most medial region was a combined of 4 map numbers (NW: *n* = 40 images; SW: *n* = 62 images), middle region was a combined of 4 map numbers (NW: *n* = 32 images; SW: *n* = 46 images) and most lateral region was a combined of 5 map numbers (NW: *n* = 52 images; SW: *n* = 56 images). This sub-regional comparison method also applies to all subsequent figure panels. *N* = 5-24 images per map number. (***B***) Analysis of the right hemisphere in NW revealed that lateral sections had significantly higher density than the medial sections (NW-R, black *p* value; *n* = 14 images for medial, 15 images for lateral). In the left hemisphere, NW-L did not show any significant difference in medial-lateral axis (grey *p* value; *n* = 8 images for medial, 13 images for lateral). Across the subregions in the medial-lateral axis, there was no significant difference between the hemispheres in NW (blue lines; medial-NW: *n* = 18 images for L, 22 images for R; middle-NW: *n* = 15 images for L, 18 images for R; lateral-NW: *n* = 26 images per hemisphere). (***C***) A different pattern of hemisphere-specific differences was observed in SW. SW-R did not have significant differences between medial and lateral sections, while SW-L did (grey *p* value denotes SW-L: *n* = 20 images for medial, 17 images for lateral; black *p* value denotes SW-R: *n* = 17 images for medial, 20 images for lateral). While PNN density did not differ between SW-L and SW-R in middle and lateral regions, SW-R exhibited significantly higher PNN density in the medial regions than SW-L (medial-SW: *n* = 28 images for L, 34 images for R; middle-SW: *n* = 24 images for L, 22 images for R; lateral-SW: *n* = 30 images for L, 26 images for R). For A-C, lines represent the mean values. Each dot represents PNN density in an individual section. *Mann-Whitney test*: *p < 0.05, n.s. = not significant, 5 mice per condition. For B and C, *n* = 1–13 images per map number.

**Figure 4.**
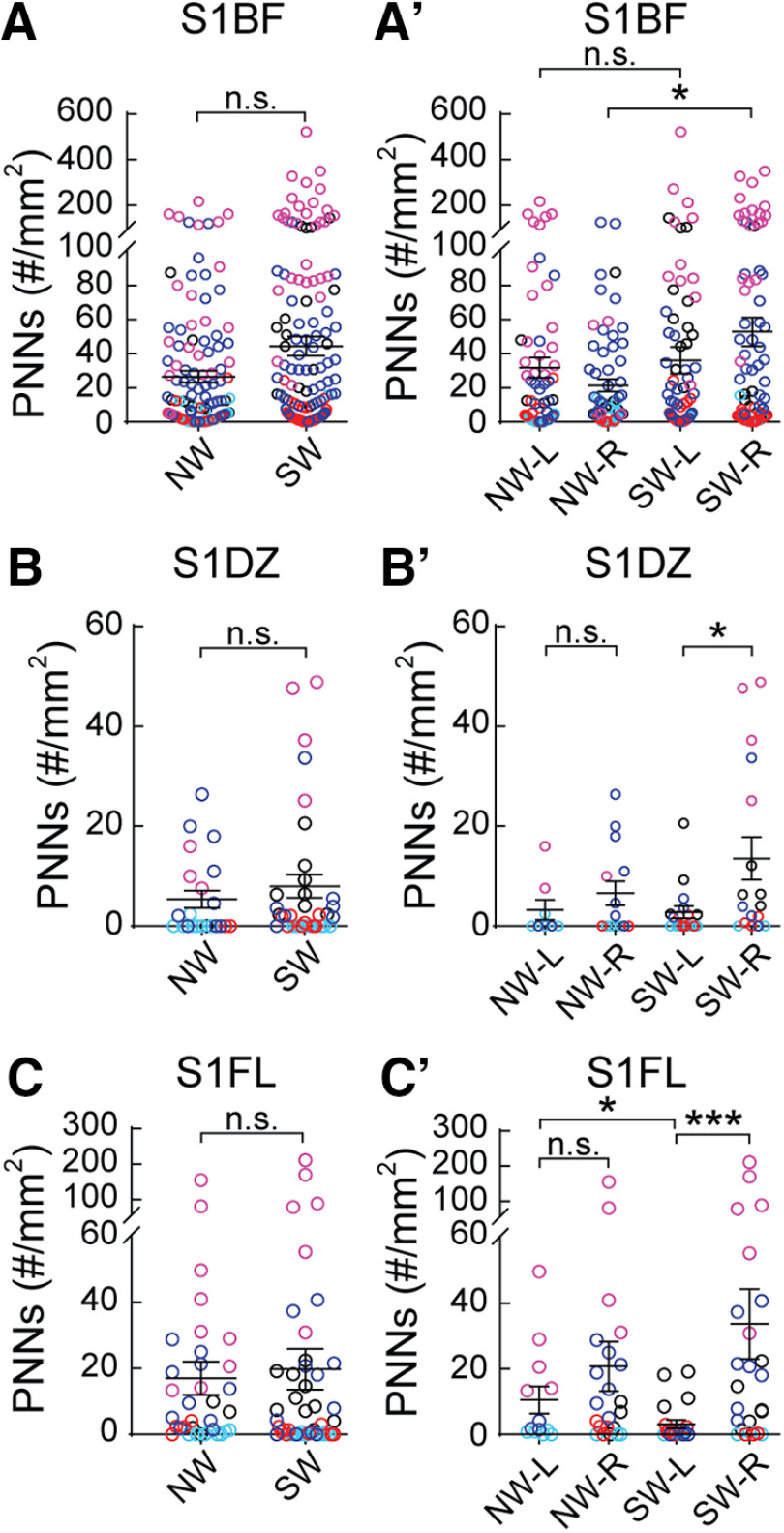
PNN density varies across subregions of WT SS1. (***A–C***) Analysis of both hemispheres for SS1 barrel field (S1BF, A), dysgranular zone (S1DZ, ***B***) and forelimb (S1FL, C) revealed no significant differences between NW and SW (S1BF: NW – *n* = 131 images, SW - *n* = 168 images; S1DZ: NW − *n* = 22 images, SW – *n* = 35 images; S1FL: NW − *n* = 35 images, SW − *n* = 46 images; 5 mice per condition; *Mann-Whitney test, p* > 0.05, n.s. = not significant). (***A′–C′***) Analysis of sub regional SS1 by hemispheres revealed dynamic changes in PNN expression. In S1BF (***A′***), a significant increase of PNN expression was detected in the right hemisphere of SW (SW-R) compared to NW (NW-R) (NW-L: *n* = 65 images; NW-R: *n* = 66 images; SW-L: *n* = 85 images; SW-R: *n* = 83 images). In S1DZ (***B′***), while no significant difference was observed in PNN density between hemispheres in NW, there was a significant increase of PNN density in the right hemisphere (SW-R) compared to the left (SW-L) (NW-L: *n* = 8 images; NW-R: *n* = 14 images; SW-L: *n* = 18 images; SW-R: *n* = 17 images). A similar pattern of PNN plasticity was also detected in S1FL (***C′***), where PNN density was significantly increased in the right hemisphere than the left of SW. Interestingly, SW-L exhibited significantly lower PNN density compared to NW-L (Figure 4C′; NW-L: *n* = 13 images; NW-R: *n* = 22 images; SW-L: *n* = 21 images; SW-R: *n* = 25 images). For ***A′-C′***, 5 mice per condition; Kruskal-Wallis followed by Dunn’s test, *p < 0.05, ***p < 0.001, n.s. = not significant.

PNN expression is predominantly found in deeper Layers IV, V, and VI in the adult SS1 in both NW (Fig. 2*A*) and SW mice (Fig. 2*B*,*C*). Across the five cohorts, PNN density (as measured by high-intensity PNN counts over area) was not significantly different between NW and SW across the whole SS1 (Fig. 2*D*). However, we noticed a wide range of distribution of PNN density within individual animals across the cohorts (Fig. 2*D*). In order to determine the source of such variability, we parsed the data according to hemispheres (Fig. 2*E*), across the lateral-medial axis (Fig. 3) and by subregions (Fig. 4). No significant difference was observed in PNN density between the left and right hemispheres of NW (Fig. 2*E*); however, the right hemisphere of SW (SW-R) had higher PNN density than the left hemisphere (SW-L; Fig. 2*B*,*C*,*E*), suggesting that higher PNN density in the SW-R SS1 might consolidate new tactile information related to the pups and/or the mother.

PNN density is PNN expression counts over area. Overall, SS1 area of the SW is significantly increased, compared with NW (Fig. 2*F*); however, that increase was not specific to a particular hemisphere within experimental condition (NW-L vs NW-R; SW-L vs SW-R; Fig. 2*G*). There is a small but significant area increase in SW-L, compared with left hemisphere of NW (Fig. 2*G*). Thus, the increased PNN density in the SW-R is mainly due to the PNN expression, and not due to the change in area.

Next, we plotted PNN density in individual sections across the lateral-medial axis (Fig. 3). We observed the individual variability in sections across map numbers in both NW and SW (Fig. 3*A*). In line with Figure 2*D*, we did not see any significant differences between NW and SW in the medial-lateral axis (light blue lines across multiple map number subdivisions). However, PNN density is significantly higher in the two most lateral sections compared with the two most medial sections within conditions (gray *p* value for NW, black *p* value for SW). Furthermore, NW had similar PNN density between left (gray) and right (black) hemispheres across the medial-lateral axis (Fig. 3*B*, light blue lines), with the more lateral regions of right NW having slightly but significantly higher PNN density compared with its medial regions (black *p* value). SW showed many sections across the medial-lateral axis with higher PNN density in the right hemisphere (black) than the left hemisphere (gray), particularly in the most medial sections (Fig. 3*C*, blue line). Within hemisphere, lateral regions of SW-L had significantly higher PNN density than its medial regions (gray *p* value), while there was no difference between these regions in SW-R (black *p* value). These findings indicate differential PNN expression in a position-specific, hemisphere-specific manner in the SS1 in both NW and SW.

### Changes in PNN density are SS1 subregion specific

Next, we examined whether specific subregions of SS1 were particularly plastic for PNN density between NW and SW. We observed no significant differences in PNN density in subregions S1BF, S1DZ, and S1FL when aggregating both hemispheres (Fig. 4*A–C*). For S1BF, a region well studied for whisker activity that contributes to tactile sensation, PNN density increased significantly and specifically in the right hemisphere for SW, compared with NW (Fig. 4*A’*). This result suggests that increased PNN density in the right hemisphere of S1BF could be a potential site for consolidation of tactile sensory information relevant for executing efficient pup retrieval. Contrary to the pattern in S1BF, S1DZ, and S1FL regions showed increased PNN density in the SW-R, compared with its SW-L (Fig. 4*B’*,*C’*). S1DZ has been implicated in proprioceptive functions, such as the movement of joints and stretch of muscle receptors (Chapin and Lin, 1984; Welker et al., 1984; Lee and Kim, 2012; Shin Yim et al., 2017); while S1FL is the sensory representation of the forepaw. Currently, the roles of these regions and PNN contribution in maternal behavior is unclear. Other brain regions (S1ULp, S1J, and S1) showed no changes after surrogacy, which further highlights S1BF as a potential site for learning consolidation (Table 1). In analyzing left hemisphere-specific data, there was a significant decrease in PNN density in S1FL of SW compared with NW (Fig. 4*C’*; Table 1, columns 5, 7). This is the first report, to our knowledge, of decreases in PNN density in a social behavior context in adult brains. Together, these results suggest that PNN density changes in adult females, in a hemisphere-specific and subregion-specific manner that is conducive for experience-dependent plasticity.

**Table 1:**
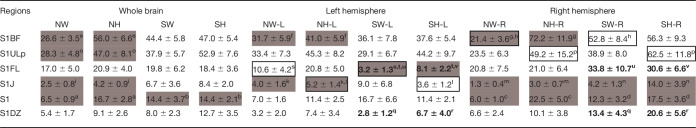
Average high-intensity PNN density across subregions and hemispheres of the SS1 before and after maternal behavior experience

Regions	Whole brain				Left hemisphere				Right hemisphere			
	NW	NH	SW	SH	NW-L	NH-L	SW-L	SH-L	NW-R	NH-R	SW-R	SH-R
S1BF	26.6 ± 3.5^e^	56.0 ± 6.6^e^	44.4 ± 5.8	47.0 ± 5.4	31.7 ± 5.9^f^	41.0 ± 5.9^f^	36.1 ± 7.8	37.6 ± 5.4	21.4 ± 3.6^g,h^	72.2 ± 11.9^g^	52.8 ± 8.4^h^	56.3 ± 9.3
S1ULp	28.3 ± 4.8^o^	47.0 ± 8.1^o^	37.9 ± 5.7	52.9 ± 7.6	33.4 ± 7.3	45.3 ± 8.2	29.1 ± 6.7	44.2 ± 9.7	23.5 ± 6.3	49.2 ± 15.2^p^	38.9 ± 8.0	62.5 ± 11.8^p^
S1FL	17.0 ± 5.0	20.9 ± 4.0	19.8 ± 6.2	18.4 ± 3.6	10.6 ± 4.2^s^	20.8 ± 5.0	**3.2 ± 1.3^s,t,u^**	**8.1 ± 2.2^t,v^**	20.8 ± 7.5	21.0 ± 6.4	**33.8 ± 10.7^u^**	**30.6 ± 6.6^v^**
S1J	2.5 ± 0.8^i^	4.2 ± 0.9^i^	6.7 ± 3.6	8.4 ± 2.0	4.0 ± 1.6^k^	5.2 ± 1.4^k,l^	9.0 ± 6.8	3.6 ± 1.2^l^	1.3 ± 0.4^m^	3.0 ± 0.7^m^	4.2 ± 1.3^n^	14.0 ± 3.9^n^
S1	6.5 ± 0.9^a^	16.7 ± 2.8^a^	14.4 ± 3.7^b^	14.4 ± 2.1^b^	7.0 ± 1.6	11.4 ± 2.5	16.7 ± 6.6	11.4 ± 2.1	6.0 ± 1.0^c^	22.5 ± 5.0^c^	12.3 ± 3.2^d^	17.5 ± 3.6^d^
S1DZ	5.4 ± 1.7	9.1 ± 2.6	8.0 ± 2.3	12.7 ± 3.5	3.2 ± 2.0	7.4 ± 3.4	**2.8 ± 1.2^q^**	**6.7 ± 4.0^r^**	6.6 ± 2.4	10.1 ± 3.8	**13.4 ± 4.3^q^**	**20.6 ± 5.6^r^**

NW, NH, SW and SH are the four different conditions. Primary somatosensory cortex subregions: S1BF, S1ULp, S1FL, S1J, S1, and S1DZ. Significant differences are denoted between genotypes by shading (E.g., NW vs NH), between hemispheres of the same condition by bold lettering, and between Naïve and Surrogate in the same genotype by bold borders. Each letter pair corresponds to statistically significant differences between two conditions. Numbers correspond to average PNN density with standard error mean across multiple sections. N = 8–85 images for hemisphere analysis, 123-168 images for combined hemisphere analysis; 5 mice per condition; *Kruskal-Wallis followed by Dunn’s test*.

### Appropriate PNN expression in SS1 is dependent on MECP2

**Figure 5.**
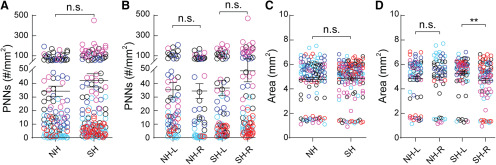
Hemisphere-specific PNN density changes are not conserved in *Mecp2^Het^* after maternal behavior. (***A***) Combined hemispheric analysis of PNN density of SS1 did not reveal significant changes between naïve Het (NH, *n* = 123 images) and surrogate Het (SH, *n* = 162 images) (*Mann-Whitney test, p > 0.05*). (***B***) Analysis of PNN density between hemispheres of SS1 revealed no significant difference between conditions or within hemispheres of naïve or surrogate Het (*n* = 54–82 images; *Kruskal-Wallis followed by Dunn’s test, p* > 0.05). (***C***) Analysis of SS1 area in both hemispheres revealed no significant changes after maternal learning (NH: *n* = 123 images; SH: *n* = 162 images; *Mann-Whitney test, p* > 0.05). (***D***) Area analysis by hemispheres reveal that left hemisphere of SH (SH-L) was significantly larger than the right hemisphere of SH (SH-R). This hemispheric area bias was absent in NH (*n* = 54–82 images; Kruskal-Wallis followed by Dunn’s test, **p < 0.01). n.s. = not significant. 5 mice per condition.

MECP2 is thought to regulate experience-dependent plasticity mechanisms in an epigenetic manner, in early postnatal development and in adulthood (Guy et al., 2001; Dani et al., 2005; Muotri et al., 2010; Cohen et al., 2011b; Noutel et al., 2011; Durand et al., 2012; Gabel et al., 2015; Krishnan et al., 2015, 2017; Lagger et al., 2017; Morello et al., 2018; Picard and Fagiolini, 2019). We previously tested this hypothesis, using an alloparental care paradigm, and found that *Mecp2*-Het adult females were inefficient at pup retrieval (Krishnan et al., 2017). In this study, we identified atypical and transient increases in PNN density in the auditory cortex of SH, leading to altered responses of PV+ neurons to auditory cues in SH (Lau et al., 2020). Here, we sought to determine whether SS1 of SH exhibited similar alterations in PNN density in subregion-specific ways. Comparing NH to SH, we noticed no significant differences in PNN density in whole SS1 (Fig. 5*A*, similar to WT in Fig. 2*D*), or within hemispheres of SS1 (Fig. 5*B*, unlike WT in Fig. 2*E*). There were no significant changes in SS1 area between NH and SH (Fig. 5*C*, unlike SW in Fig. 2*F*). However, an increased SS1 area in left versus right hemisphere of SH was noted (Fig. 5*D*).

**Figure 6.**
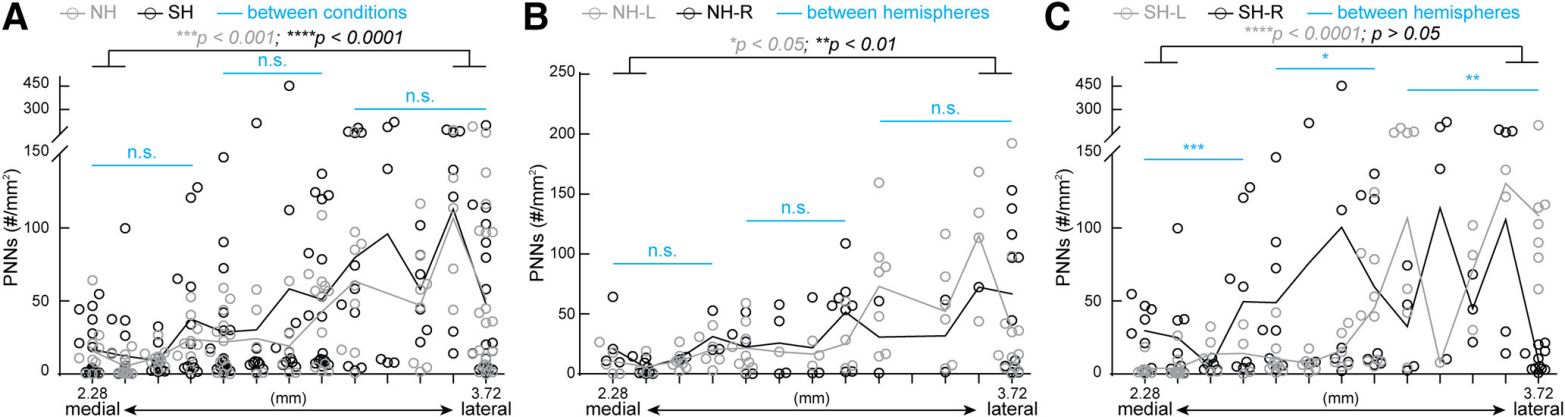
Overall patterns of PNN density across medial-lateral axis is preserved in *Mecp2^Het^*. (***A***) Similar to NW-SW in Figure 3, NH and SH displayed significant increase of PNN density in lateral sections compared to the medial sections of the same condition (grey *p* value denotes NH: *n* = 23 images for medial, 27 images for lateral; black *p* value denotes SH: *n* = 36 images for medial, 25 images for lateral). Along the sub regions encompassing the medial-lateral axis, there was no significant difference between NH and SH, represented by the light blue lines (medial: *n* = 42 images for NH, 59 images for SH; middle: *n* = 37 images for NH, 55 images for SH; lateral: *n* = 44 images for NH, 48 images for SH). *N* = 5–22 images per map coordinate. (***B***) Within hemispheres of NH, statistical analysis of the lateral sections of the right (NH-R, black *p* value) and the left (NH-L, grey *p* value) showed significantly higher PNN density than their medial sections (NH-L: *n* = 9 images for medial, 16 images for lateral; NH-R: *n* = 14 images for medial, 11 images for lateral). There was no significant difference between hemispheres along the medial-lateral axis (medial: *n* = 23 images for L, 19 images for R; middle: *n* = 17 images for L, 20 images for R; lateral: *n* = 29 images for L, 15 images for R). (***C***) A different pattern of hemisphere-specific differences was observed in SH. SH-R did not display significant difference between medial and lateral sections, while SH-L did (grey *p* value denotes SH-L: *n* = 9 images in lateral, *n* = 20 images for medial; black *p* value denotes SH-R: *n* = 16 images for both lateral and medial). Statistical comparison between hemispheres revealed dynamic differences across medial-lateral axis, with SH-R had significantly higher PNN density than SH-L in medial and middle regions, while SH-L had significantly higher PNN density in the lateral regions (light blue lines; medial: *n* = 29 images for L, 30 images for R; middle: *n* = 31 images for L, 24 images for R; lateral: *n* = 20 images for L, 28 images for R). For ***A–C***, lines represent mean values. Each dot represents PNN density in an individual section. Mann-Whitney test: *p < 0.05, **p < 0.01, ***p < 0.001, ****p < 0.0001, n.s. = not significant, 5 mice per condition. For ***B–C***, *n* = 1–12 images per map coordinate.

For PNN density analysis across medial-lateral axis (Fig. 6), PNN density was similar between NH and SH along this axis (Fig. 6*A*, light blue lines), with significantly more PNNs in the lateral regions than the medial regions within conditions (gray *p* value for NH, black *p* value for SH). Within NH, there was no difference between left and right hemispheres along medial-lateral axis (Fig. 6*B*, light blue lines). Again, lateral regions expressed significantly more PNNs than the medial regions within each hemisphere (gray *p* value for NH-L, black *p* value for NH-R). After surrogacy, SH exhibited significantly more dynamic changes in PNN density across medial-lateral axis between left and right hemisphere (Fig. 6*C*, light blue lines). Moreover, while PNN densities in lateral and medial regions were not significantly different in SH-R (black *p* value), lateral regions of SH-L had significantly more PNNs than the its medial regions (gray *p* value).

**Figure 7.**
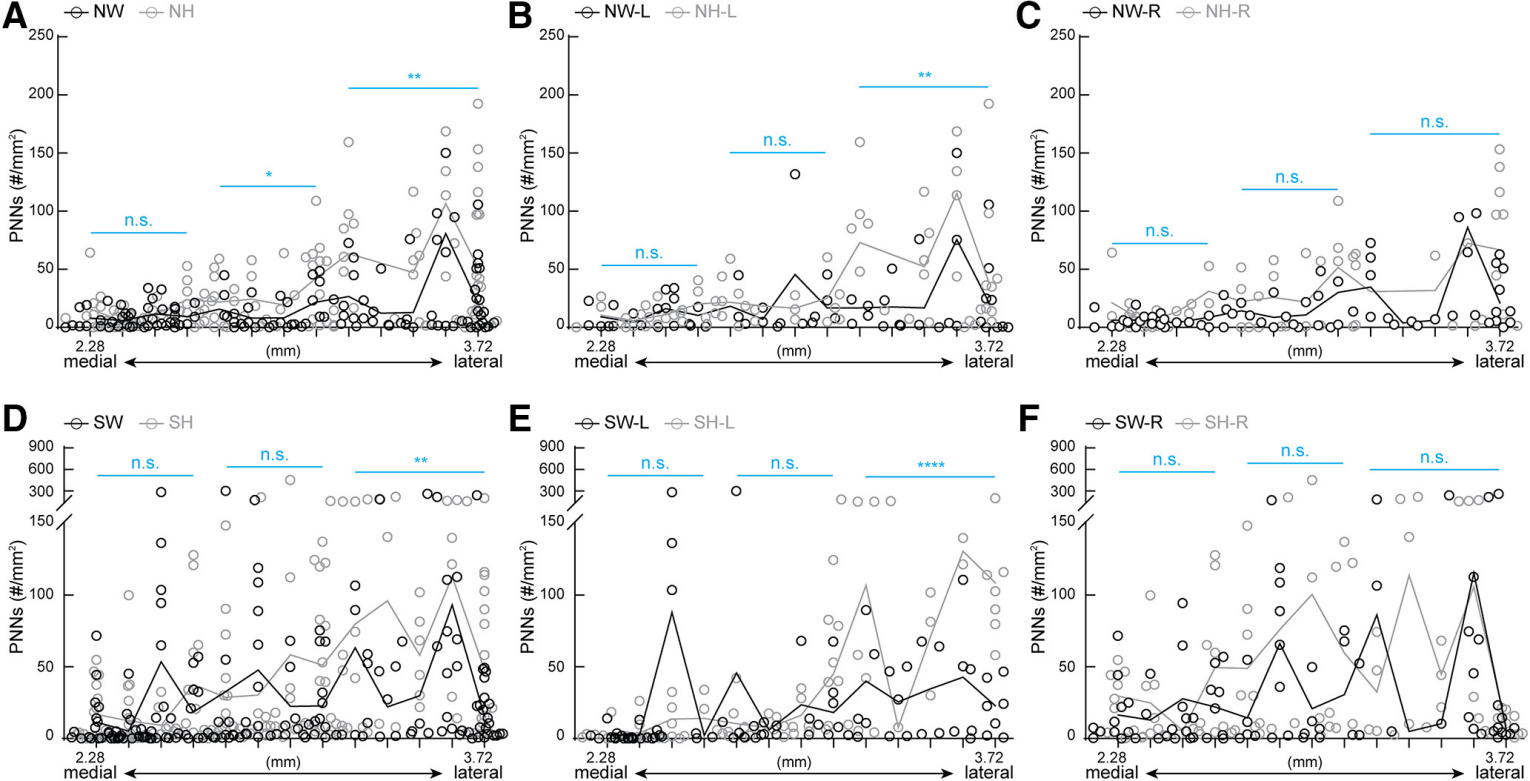
Left hemispheres of naïve and surrogate *Mecp2^Het^* have higher PNN density in the most lateral sections, compared to the wildtype counterparts. (***A***) Combined hemispheres of NH had significantly higher PNN density than NW, in the middle and lateral sections. No significant difference was observed in the most medial sections (medial: *n* = 40 images for NW, 42 images for NH; middle: *n* = 32 images for NW, 37 images for NH; lateral: *n* = 52 images for NW, 44 images for NH). (***B***) Comparing left hemisphere PNN density between NW and NH, NH had significantly higher PNN density in the most lateral sections, with no significant differences in the more medial sections (medial: *n* = 18 images for NW-L, 23 images for NH-L; middle: *n* = 15 images for NW-L, 17 images for NH-L; lateral: *n* = 26 images for NW-L, 29 images for NH-L). (***C***) There were no significant differences in the right hemisphere between NW and NH, suggesting the PNN expression in the subregions of the lateral sections of the left hemisphere are particularly dysregulated in NH (medial: *n* = 22 images for NW-R, 19 images for NH-R; middle: *n* = 18 images for NW-R, 20 images for NH-R; lateral: *n* = 26 images for NW-R, 15 images for NH-R). (***D***) SH exhibited higher PNN density over SW only in the most lateral regions (medial: *n* = 62 images for SW, 59 images for SH; middle: *n* = 46 images for SW, 55 images for SH; lateral: *n* = 56 images for SW, 48 images for SH). (***E***) Similar to naïve conditions, only the lateral sections in left hemisphere of SH had significantly higher PNN density than the same regions of SW (medial: *n* = 28 images for SW-L, 29 images for SH-L; middle: *n* = 24 images for SW-L, 31 images for SH-L; lateral: *n* = 30 images for SW-L, 20 images for SH-L). (***F***) No significant differences between SW and SH were found in the right hemisphere (medial: *n* = 34 images for SW-R, 30 images for SH-R; middle: *n* = 22 images for SW-R, 24 images for SH-R; lateral: *n* = 26 images for SW-R, 28 images for SH-R). For A-F, lines represent the mean values. Each dot represents PNN density in an individual section. *Mann-Whitney test*, *p < 0.05, **p < 0.01, ****p < 0.0001, n.s. = not significant. 5 mice per condition. For ***A*** and ***D***, *n* = 5-24 images per map coordinate. For ***B–C*** and ***E–F***, *n* = 1–13 images per map coordinate.

**Figure 8.**
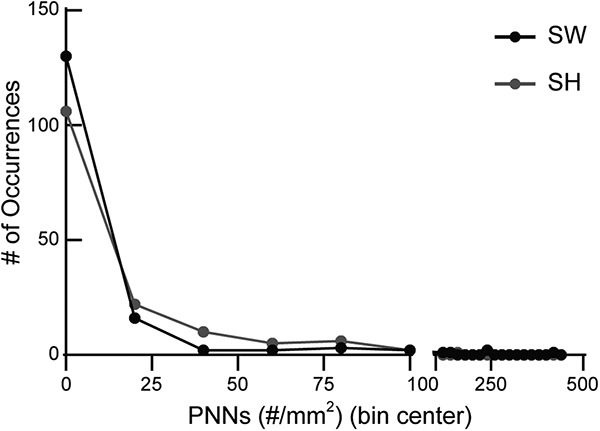
Significant difference in S1 PNN density between SH and SW in Table 1 due to differential distribution. Histogram analysis showed that SW had more occurrences of 0 PNN density compared to SH, as shown by black dot (SW) above grey dot (SH). SH had more occurrences of 20–80 PNN density than SW, as shown by grey dots (SH) above black dots (SW). The average PNN density values of SW (*n* = 160 images) and SH (*n* = 152 images) are similar (Table 1) (5 mice per condition), but they are statistically different due to this differential distribution of data.

When we compared PNN density between genotypes, WT and Het (Table 1; Fig. 7), we noticed significant hemisphere-specific and subregion-specific differences, with the maintenance of medial-lateral axis. NH had increased PNN density over NW across medial-lateral axis (Fig. 7*A*) and significant differences between genotypes after surrogacy (SW vs SH) in lateral sections (Fig. 7*D*, light blue line). S1 was an exception, which showed statistical significance, though the mean density was similar (Table 1). This is likely due to differential distribution of PNN density (Fig. 8).

In the left hemisphere, NH and SH (Fig. 7*B*,*E*, respectively) had significantly higher PNN density in the lateral sections, compared with NW and SW, respectively. No significant differences were observed between genotypes in the right hemisphere (Fig. 7*C*,*F*). While analyzing subregions, in the left hemisphere, NH had increased PNN density in S1BF and S1J, compared with NW (Table 1, columns 5, 6). SH had increased PNN density in only S1FL, compared with SW (Table 1, columns 7, 8). In the right hemisphere, NH had increased PNN density in S1BF, S1J, and S1, compared with NW. SH had increased PNN density in S1J and S1, compared with SW. Surrogacy correlated with higher PNN density in only right hemisphere of WT S1BF (NW-R vs SW-R; also Fig. 2*E*), while this was not seen in Het (NH vs SH), with NH-R already exhibiting high PNN densitycompared with NW-R (Table 1, columns 9, 10). S1ULp showed higher PNN density in SH-R compared with NH-R (Table 1, columns 10, 12), which was not observed in WT. Taken together, these results suggest that MECP2 regulates dynamic PNN expression, which is then important for appropriate maternal behavior.

Comparing the hemispheres within genotypes, NW and NH did not have significant differences in PNN density in subregions. However, PNN density increased in the right hemisphere of S1FL and S1DZ in both SW and SH (Table 1) compared with SW-L and SH-L, respectively. These results suggest a preserved common experience-dependent plasticity mechanism activation in these regions within the right hemisphere across genotypes in similar social contexts.

### PCA identifies lateral-medial and hemisphere-specific changes in PNN expression

Because of the increasing number of variables being compared, we chose an unsupervised statistical procedure called Principal Component Analysis (PCA), commonly used in genomics/transcriptomics analysis, to determine whether patterns emerge from the PNN density data. PCA takes a set of measurements across samples and identifies the measurements that best capture the variation among the samples. It results in a set of uncorrelated components (called principal components) that each capture an orthogonal aspect of the differences across the samples. As input to PCA, we used PNN densities across individual sections and map numbers (represented as lateral coordinates) across all conditions in the five cohorts. If multiple sections per map number were present, values were averaged across sections to give a single density value.

**Figure 9.**
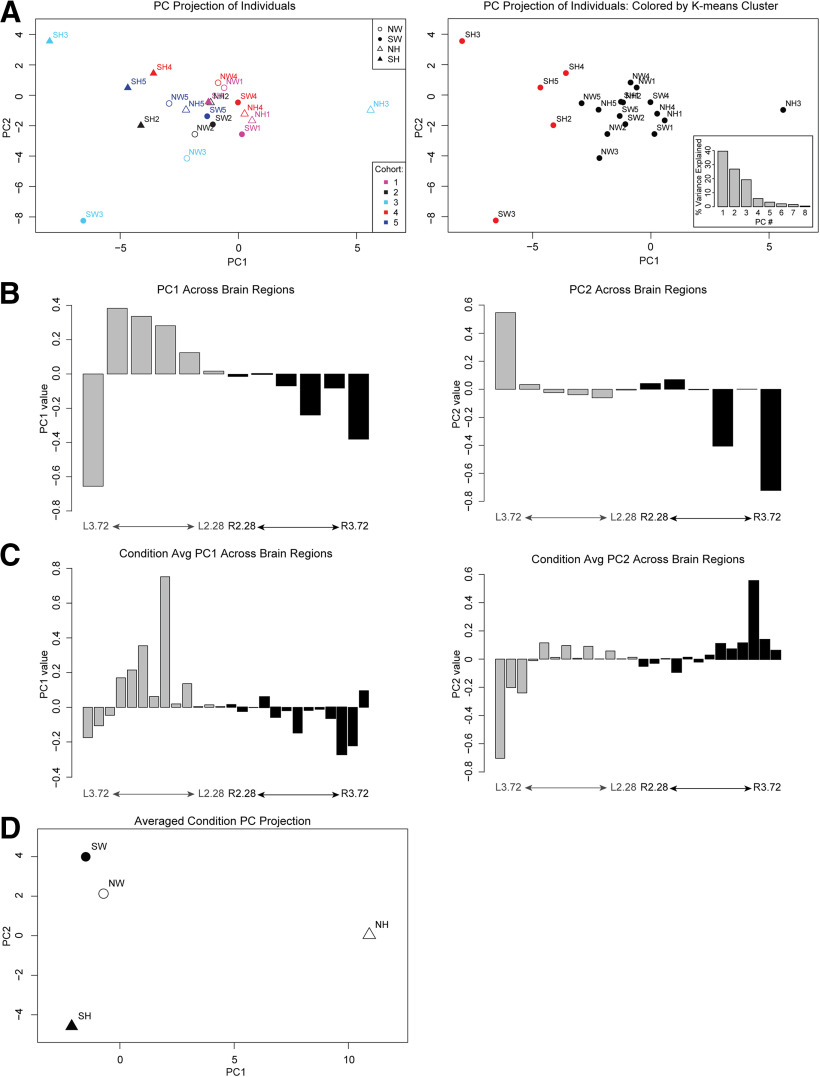
Principal component analysis of PNN expression segregates by conditions, lateral/medial axis and hemispheres. (***A***) The projection of each individual brain onto PC1 and 2. These PCs are defined by relative weights of the different brain regions – all of the weights for PC1 and 2 are shown in ***B***. (Left) Individuals are colored by cohort with symbol shapes corresponding to their genotype and experience condition. (Right) Individuals are colored by K-means clustering assignment, showing that the primary separation is SH from the rest of the conditions. While PC1 tends to distinguish surrogates from naïve (in particular, surrogate het from all others), the PCA itself is an unbiased analysis that is not designed to calculate the differences in “NH vs. SH”. Rather, the PC1 represents the combination of factors that are the largest source of variation among all the samples. The PC1 itself is fully defined by the weights of the different brain sections. Inset shows the % variance explained by the different PCs, with PC1 explaining the most variance. (***B***) Weights for each brain region for principal component (PC) 1 (left panel) and 2 (right panel) from the analysis in (***A***). The map regions (corresponding lateral coordinates) with strongest positive and negative values contribute most strongly to the variation between individuals. The weights show a trend from medial to lateral, showing the differences in laterality are the largest source of variation in the data. (***C***) As in ***B***, weights for each brain region for PC1 (left panel) and 2 (right panel) are shown, in this case for PCA on data in which all cohorts were averaged for each condition. (***D***) Conditions projected onto PC1 show a separation of NH from the rest while PC2 axis shows a separation of SH from SW.

In the first analysis (Fig. 9*A*,*B*), we sought to determine whether the PNN patterns segregated primarily by cohort or condition (genotype and experience). We preserved data for each individual brain and performed PCA on PNN densities averaged across every set of two adjacent map regions. By examining the projection of each individual onto the first and second principal components, we found that, while there is biological variability between cohorts, the individuals in a given cohort did not cluster separately from one another in this unsupervised analysis (Fig. 9*A*, left), suggesting that technical variability in processing samples across five cohorts is not the primary driver of PNN density differences. This is an important control to assess technical or biological variability in this data. Instead, the first principal component (PC1), which explains 40% of the variation in the data (Fig. 9*A*, right, inset), distinguished the SH PNN density patterns from all others, especially NH (Fig. 9*A*, right). We then examined the weights of each brain region in PC1 to determine which regions are most important for capturing the differences between SH and NH. The weights of each section in PC1 (and PC2) shows a left-right asymmetry and increasing weights for lateral versus medial sections, confirming our previous observations (Fig. 9*B*). The findings from our first PCA confirms that variations among mice are resulting from genetic and/or environmental differences and not technical biases. The results further validate our previous observations of asymmetry in PNN density of left and right hemispheres, augmentation of PNN density in lateral sections, and altered SH PNN density patterns.

In the second PCA (Fig. 9*C*,*D*), we sought to determine the major PNN density patterns that distinguish genotypes and conditions. Instead of averaging map numbers as in the previous analysis, we preserved data for each map number and averaged across the five cohorts for each condition and then performed PCA to determine the major distinguishing patterns. This analysis revealed patterns of PNN density that best distinguish NH versus SH (PC1) and SW versus SH (PC2; Fig. 9*C*,*D*). These PC patterns also reflect the medial/lateral and left/right asymmetries, thus confirming the anatomic and neurobiological distinctions in the previous figures. Overall, we observe that unsupervised analysis identifies these lateral/medial and left/right asymmetries as the major pattern that characterizes the differences in PNN distribution between experience and genotype.

### Individual mice exhibit strong laterality for PNN expression

**Figure 10.**
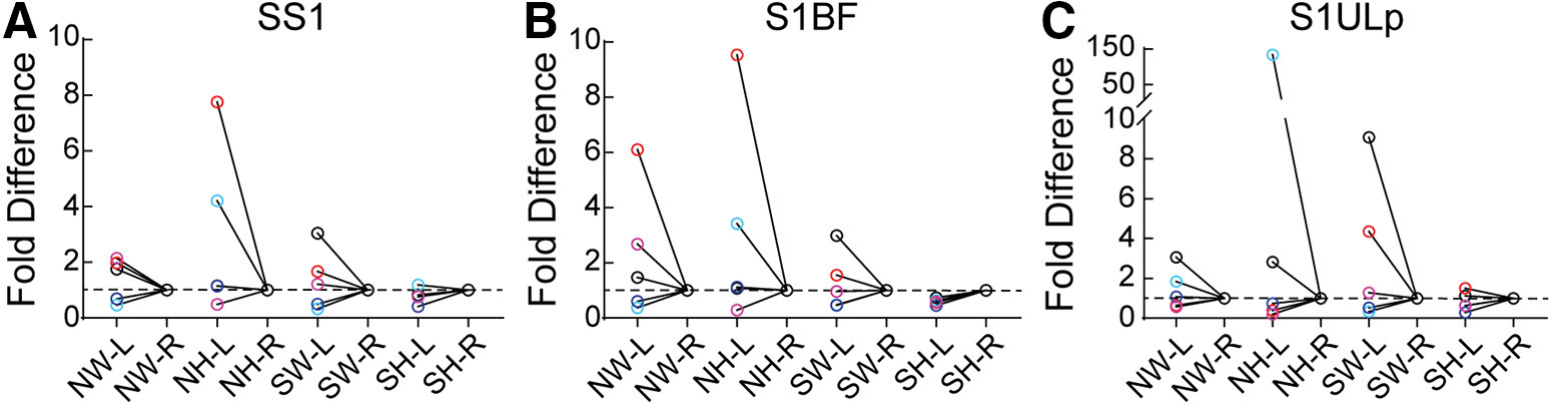
Individual brains exhibit differential hemispheric bias in PNN density in specific subregions of SS1. (***A–C***) For each brain, PNN density from the left hemisphere was normalized to the right hemisphere. This left-right hemisphere normalization revealed varying patterns of cortical asymmetry. (***A***) In SS1, three NW brains exhibit left hemisphere bias of 2-fold. In SW, left hemisphere bias is seen in 2 brains (black, red). Two NH brains exhibit large fold differences favoring left hemisphere (red, blue circles). In SH, no brains display cortical asymmetry. Similar trends with larger fold differences are seen in S1BF (***B***) and S1ULp (***C***).

As the previous data were an aggregate/average of five biological replicates, we were interested in determining whether hemispheric biases in PNN density were seen in individual mice. For each mouse, we normalized PNN density of left hemisphere to the right hemisphere (Fig. 10). In SS1 as a whole, a modest left hemisphere bias was seen in three out of five mice across conditions and genotypes (Fig. 10*A*). Higher differences in left hemisphere bias is seen in subregions such as S1BF (Fig. 10*B*) and S1ULp (Fig. 10*C*). Interestingly, a decrease in the left hemisphere bias was observed in most of the SH mice (Fig. 10*A–C*), suggesting that SH brains have intact plasticity mechanisms that can be triggered by this social maternal experience to overcome the abnormal high PNN density lateralization in NH. Together, these results show that individual mice have differing hemispheric bias in PNN density in SS1, which may contribute, in a MECP2-dependent manner, to individual variability in responding to and consolidating new tasks involved in tactile sensation.

**Figure 11.**
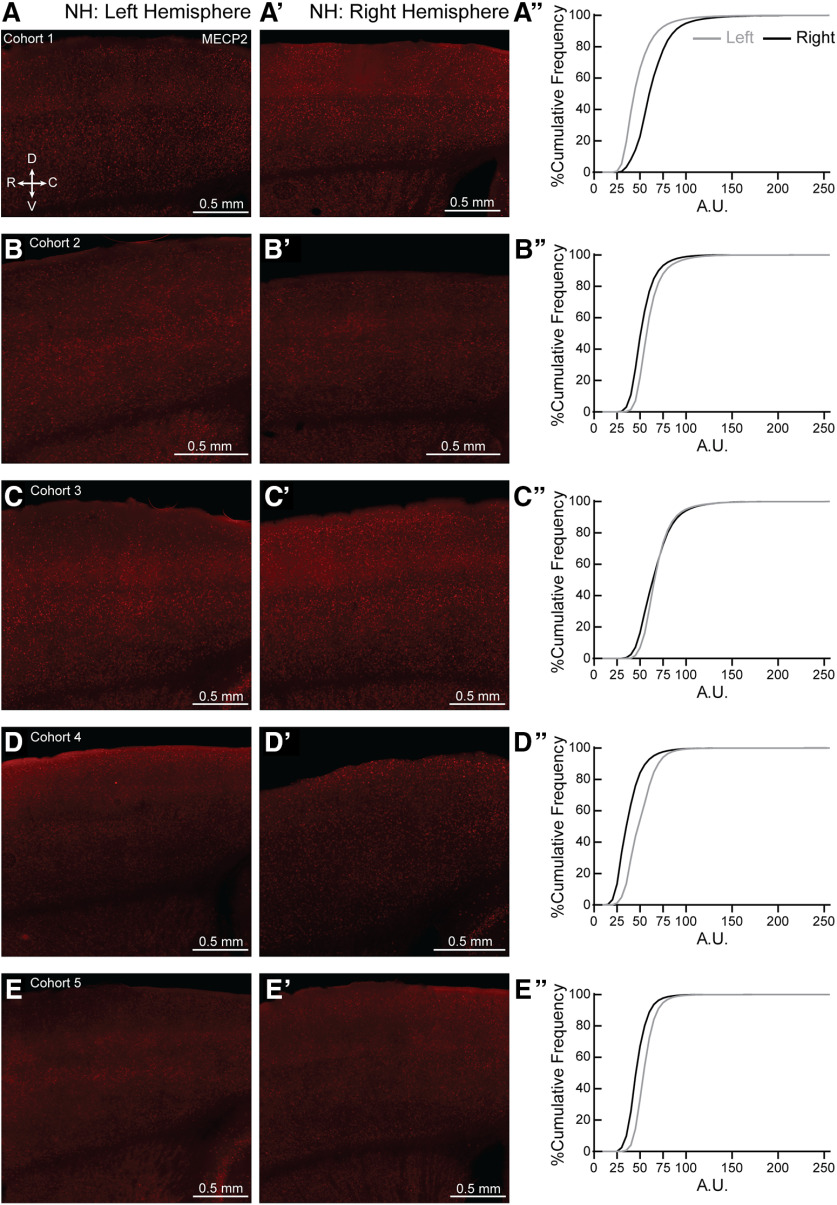
Hemispheric bias for MECP2 expression in individual NH brains. (***A–E***, ***A′–E′***) Representative 20X magnification, tiled projection epifluorescent images showing MECP2 expression in left (***A–E***) and right (***A′–E′***) SS1 of NH from cohorts 1-5. R = rostral, C = caudal, D = dorsal and V = ventral. (***A′′–E′′***) Percentage cumulative frequency distribution of MECP2 intensity within left (grey) and right (black) SS1 of the corresponding NH cohorts. Cohort 1 (***A′′***) expressed more MECP2 in the right hemisphere than the left. Cohorts 2, 4–5 (***B′′, D′′–E′′***, respectively) expressed more MECP2 in the left hemisphere than the right, while cohort 3 (***C′′***) showed similar MECP2 expression in both hemispheres. A.U. = arbitrary intensity unit. N = 1 image per hemisphere.

**Figure 12.**
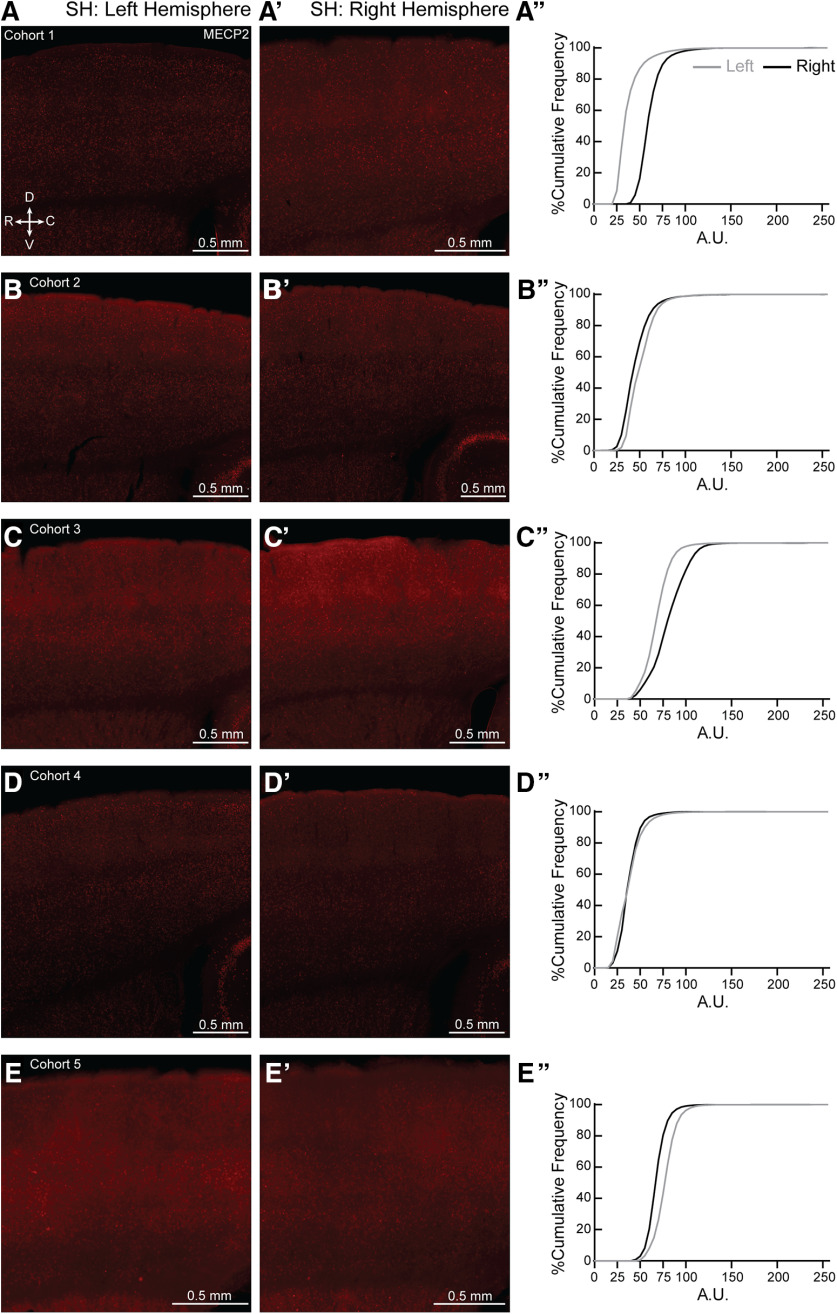
Hemispheric bias for MECP2 expression in individual SH brains. (***A–E, A′-E′***) Representative 20X magnification, tiled projection epifluorescent images showing MECP2 expression in left (***A–E***) and right (***A′-E′***) SS1 of SH from cohorts 1-5. R = rostral, C = caudal, D = dorsal and V = ventral. (***A′′-E′′***) Percentage cumulative frequency distribution of MECP2 intensity within left (grey) and right (black) SS1 of the corresponding SH cohorts. Cohort 1 and 3 (***A′′*** and ***C′′***, respectively) expressed more MECP2 in the right hemisphere than the left. Cohorts 2 and 5 (***B′′*** and ***E′′***, respectively) expressed slightly more MECP2 in the left hemisphere than the right, while cohort 4 (***D′′***) showed similar MECP2 expression in both hemispheres. A.U. = arbitrary intensity unit. N = 1 image per hemisphere.

**Figure 13.**
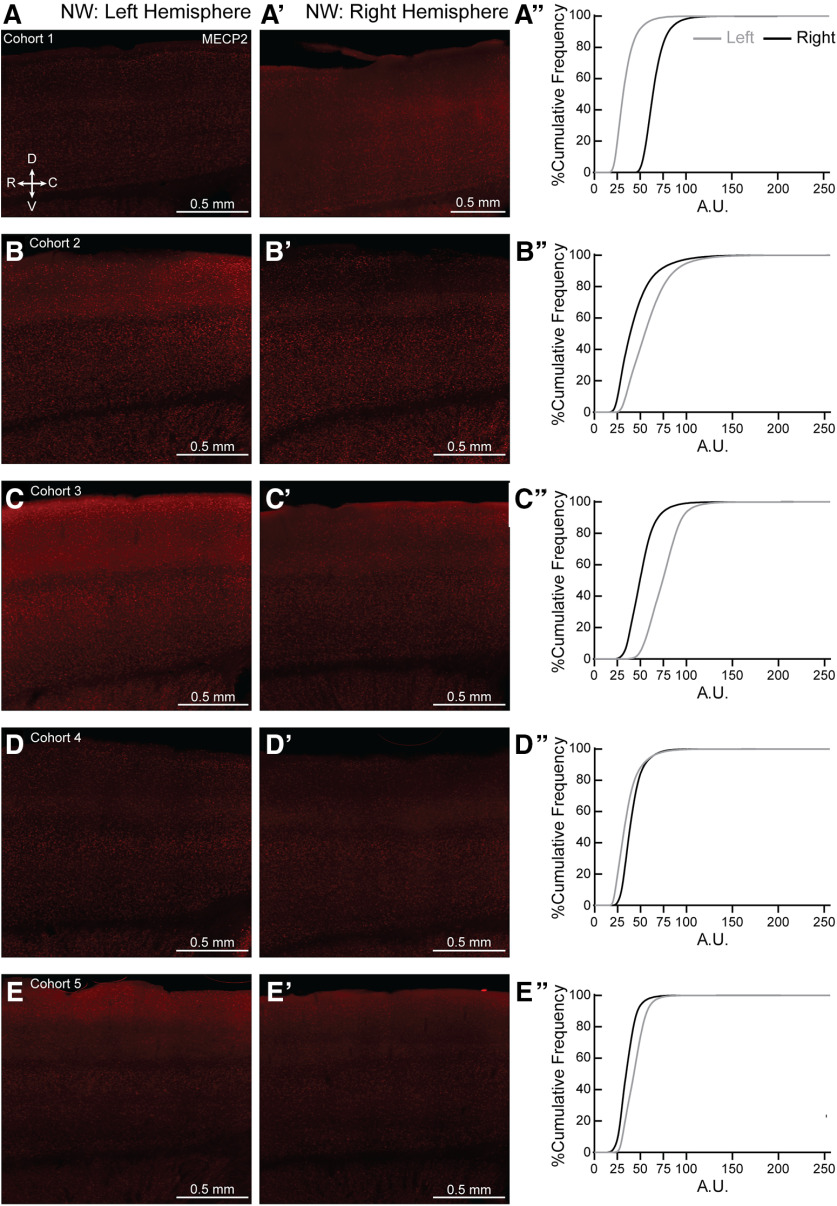
Hemispheric bias for MECP2 expression in individual NW brains. (***A–E, A′–E′***) Representative 20X magnification, tiled projection epifluorescent images showing MECP2 expression in left (***A–E***) and right (***A′–E′***) SS1 of NW from cohorts 1-5. R = rostral, C = caudal, D = dorsal and V = ventral. (***A′′–E′′***) Percentage cumulative frequency distribution of MECP2 intensity within left (grey) and right (black) SS1 of the corresponding NW cohorts. Cohort 1 and 4 (***A′′*** and ***D′′***, respectively) expressed more MECP2 in the right hemisphere than the left. Cohorts 2, 3 and 5 (***B′′***, ***C′′*** and ***E′′***, respectively) expressed more MECP2 in the left hemisphere than the right. A.U. = arbitrary intensity unit. N = 1 image per hemisphere.

**Figure 14.**
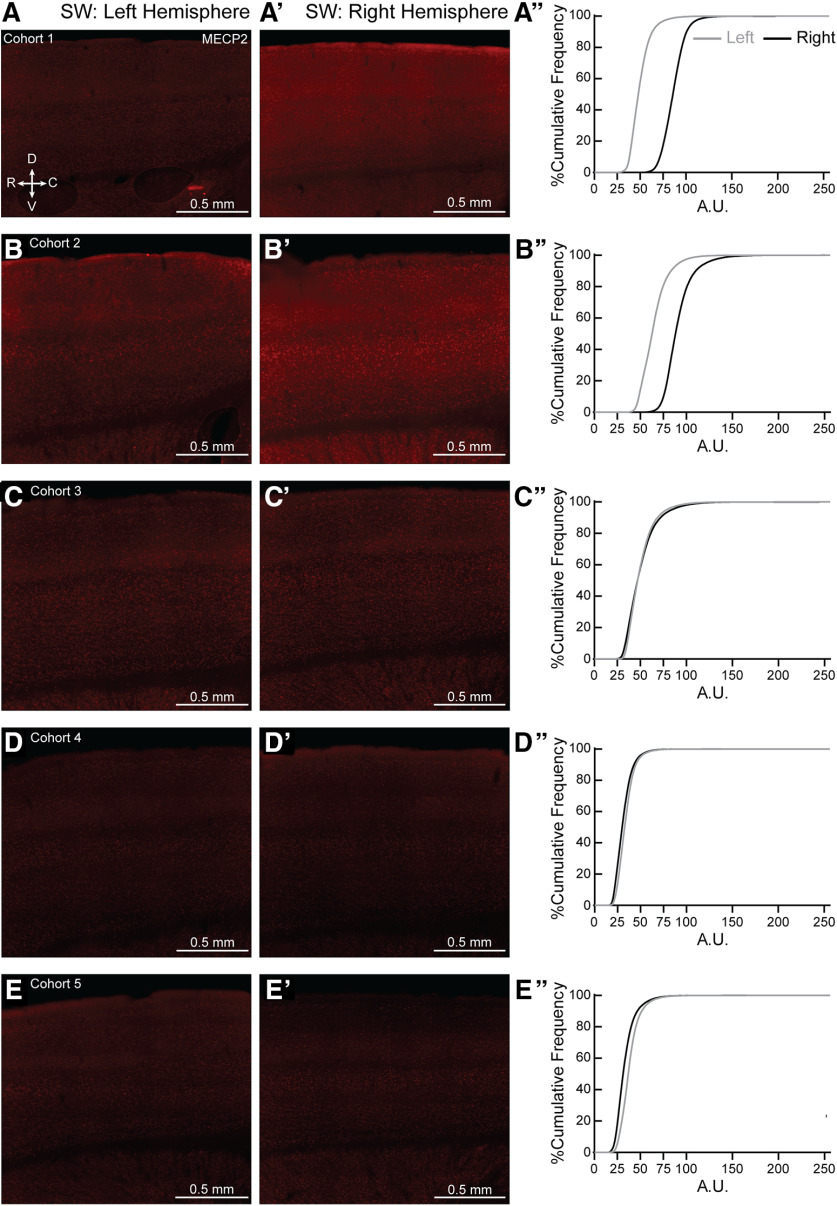
Hemispheric bias for MECP2 expression in individual SW brains. (***A–E, A′–E′***) Representative 20X magnification, tiled projection epifluorescent images showing MECP2 expression in left (***A–E***) and right (***A′–E′***) SS1 of SW from cohorts 1-5. R = rostral, C = caudal, D = dorsal and V = ventral. (***A′′–E′′***) Percentage cumulative frequency distribution of MECP2 intensity within left (grey) and right (black) SS1 of the corresponding SW cohorts. Cohort 1 and 2 (***A′′*** and*** B′′***, respectively) expressed more MECP2 in the right hemisphere than the left. MECP2 expressions in Cohorts 3-5 (***C′′-E′′***) were similar between left and right hemispheres. A.U. = arbitrary intensity unit. N = 1 image per hemisphere.

*Mecp2* is a X-linked gene. It is possible that skewed MECP2 expression could lead to the individual variability seen in the NH and SH, though this explanation does not apply to the variability seen in the WT as well. Initial observation of individual sections for all ten NH and SH brains did not show patchy, but salt-and-pepper expression of MECP2, in agreement with other work (Metcalf et al., 2006; Rietveld et al., 2015; for review, see Ribeiro and MacDonald, 2020), especially in the age of our mice used in the study (7–12 weeks old). However, we did find left-right asymmetry in MECP2 expression in both NH (Fig. 11*A’’*,*B’’*,*D’’*,*E’’*) and SH (Fig. 12*A’’*,*B’’*,*C’’*,*E’’*) brains. We also found left-right asymmetry in MECP2 expression in both NW (Fig. 13, to varying degrees in most animals) and SW (Fig. 14*A’’*,*B’’*,*E’’*). Together, these results suggest variable MECP2 expression between hemispheres in the same brain of either genotype (WT, Het) or condition (Naive, Sur).

## Discussion

Given the long-standing and revitalized interest in extracellular matrix structures in the brain, we sought to systematically characterize high-intensity PNN expression in the whole SS1 in a model of adult experience-dependent plasticity, in relevant social behavioral conditions. To our knowledge, this is the first systematic characterization of PNN expression in the adult SS1 with detailed information about subregions and laterality in individual mice.

In early postnatal cortical development, expression of PNNs increases progressively with the maturation of that network. An excellent example is the primary visual cortex where the developmental increase of PNNs is regulated by visual experience (Beurdeley et al., 2012; Hou et al., 2017). PNNs in mature primary visual cortex mainly surround the soma and proximal dendrites of PV+ interneurons (Hartig et al., 1992; Celio, 1993; Ueno et al., 2018). Mature PNNs are thought to be inhibitory for experience-dependent plasticity, as their increase in developing primary visual cortex correlates with the termination of the critical period and PNN removal in adult primary visual cortex restores plasticity, as measured by ocular dominance plasticity assays (Pizzorusso et al., 2002, 2006; Bavelier et al., 2010). These and studies in other brain regions (amygdala, hippocampus, piriform, and auditory cortex) have suggested that PNNs are stable, long-term structures (Sorg et al., 2016; Miyata and Kitagawa, 2017; Ueno et al., 2019). Experiments involving the enzymatic removal of PNN by chondroitinase ABC (ChABC) or hyaluronidase injections in amygdala, hippocampus, piriform and auditory cortices have shown that synaptic plasticity can be reactivated (Pizzorusso et al., 2002; Gogolla et al., 2009; Kochlamazashvili et al., 2010; Banerjee et al., 2017; Krishnan et al., 2017; Thompson et al., 2018).

A word of caution: due to ease of immunostaining with WFA and manipulation experiments with ChABC, many studies now employ PNNs as markers for plasticity. Our characterization in adult brains shows that these structures are dynamic and have hemisphere-specific and subregion-specific expression, hinting at potential neural circuitry mechanisms involving laterality in mice. Systematic and careful analysis must be taken to fully characterize PNN expression in experimental design rather than using standard “representative” sample approaches in immunostaining.

### What governs PNN dynamics in adults?

Matrix metallopeptides and proteases are known to assist in remodeling extracellular matrix structures (Lu et al., 2011; Miyata and Kitagawa, 2017; Bozzelli et al., 2018). However, the contexts and mechanisms for inducing remodeling in adult brains are currently unclear. Some regional and temporal changes in PNN density have been described before (Ueno et al., 2018, 2019). However, systematic, finer scale whole-brain analysis of WFA expression across entire brain regions during development and adulthood has not been performed. Our study suggests PNNs, as measured by WFA immunostaining, may not be stable and static structures as once thought. In this study, we show that high-intensity PNNs of SS1 exhibit increased and decreased expression in a subregion-specific, hemisphere-specific manner, after maternal behavior experience. Currently, these differences in PNN expression between naive and Sur occur over one to two weeks (3–5 d before pups are born plus 6 d of behavior before mice are perfused). The rate of PNN formation and remodeling, which might ultimately affect tactile perception and efficient pup retrieval, remains unknown.

**Figure 15.**
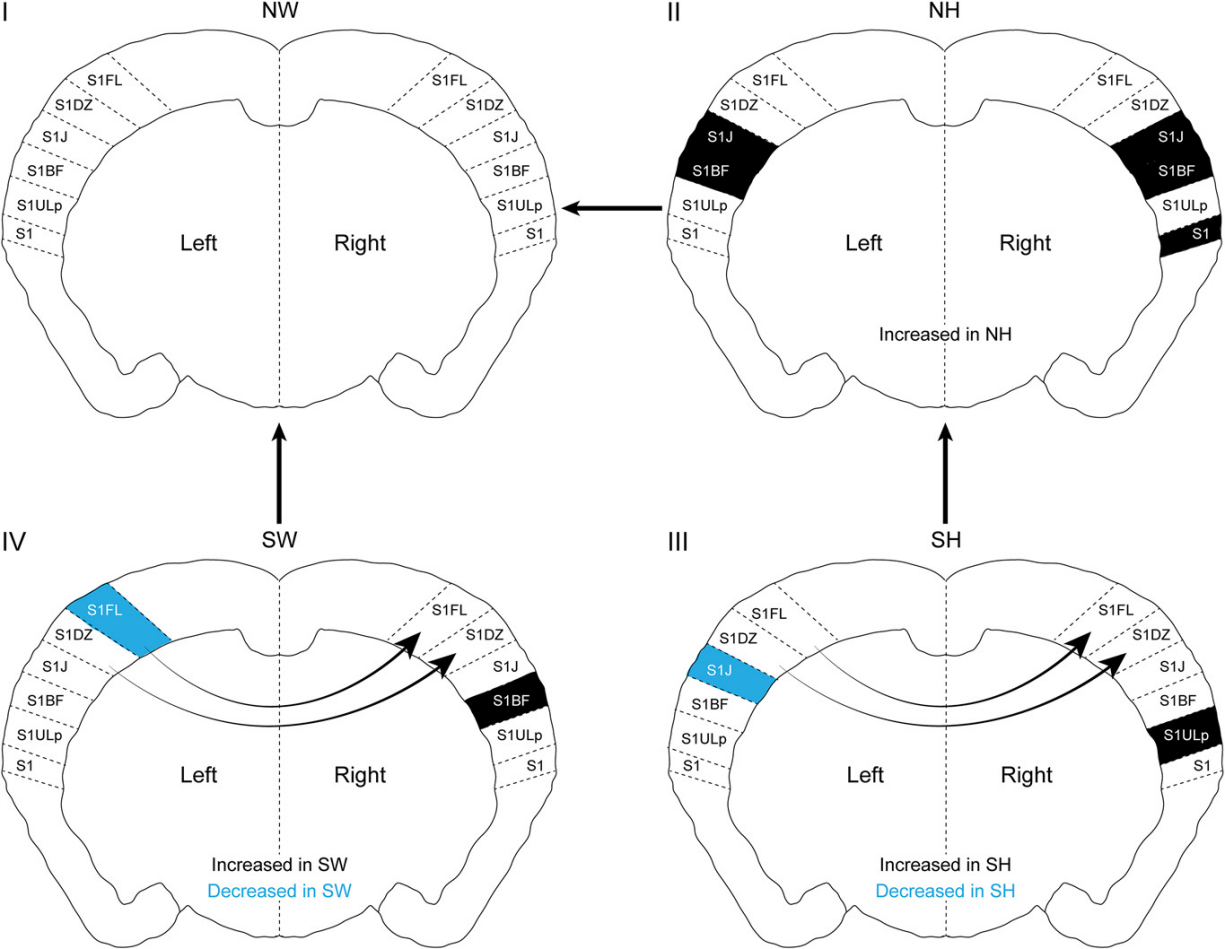
Summary of changes in PNN density between genotypes in maternal behavior context. (Quadrants I-IV) The changes in PNN density (grey and black shading) marked inside the brain slices denote comparisons between conditions connected by outside arrows between brain schemas. Arrows inside the brain schemas indicate hemispheric differences within genotype, with arrowheads pointing to the hemisphere with the higher PNN density. (IVàI) Comparing SW to NW, PNN density is increased in right S1BF (black) and decreased in left S1FL (blue) regions of SW. Within SW, PNN density is higher in the right hemisphere, particularly in S1FL and S1DZ. Taken together, PNN density changes in these particular subregions could contribute to tactile perception in SW, ultimately leading to efficient pup retrieval. (IIàI) NH has increased PNN density in specific subregions compared to the NW, suggesting possible tactile perception issues before maternal experience, which could contribute to *Mecp2^Het’^S* inefficient pup retrieval performance. (IIIàII) SH has increased PNN density in the right S1ULp (black) and decreased PNN density in left S1J (blue), compared to NH, suggesting possible compensatory plasticity mechanisms after maternal experience in *Mecp2^Het^*. SH also displays higher right hemisphere PNN density in S1FL and S1DZ than its left hemisphere, similar to SW, suggesting that right hemisphere-specific increases in PNNs in S1FL and S1DZ might be important for processing tactile information during pup retrieval task.

In the Paxinos and Franklin atlas, anatomic subregions were classified based on structural connectivity studies. Based on these anatomic characterizations, we speculate that changes in PNN density in specific subregions could impact information processing. For example, when NW female mice learn maternal behavior to become Sur, there is higher PNN density in the right S1BF, with a concomitant lower PNN density in left S1FL (Fig. 15, colors within IV), suggesting that these changes contribute to solidifying new synaptic contacts in S1BF, while promoting remodeling in S1FL. Together, these changes could ultimately help process new tactile information related to pups and the mother acquired by the whiskers and forelimbs. Furthermore, right hemisphere specific PNN increases in S1FL and S1DZ suggests specific rewiring in subregions that could contribute to efficient information processing associated with laterality and dominant hemispheres (Fig. 15, arrows within IV). The observed fine-scale changes in PNN expression in the adult SS1 before and after maternal behavior experience suggest specific hypotheses about connectivity and functional changes in subregions, specifically in barrel field and upper lip subregions of both hemispheres*. In vivo* electrophysiological and/or imaging studies, which measure dynamics of neural circuitry activation and processing in intact brains, would help prove these hypotheses.

This idea of lateralization3 of function in the rodent brain has been a topic of longstanding yet of sporadic interest in the field (Glick and Ross, 1981; Kim et al., 2012 Lu et al., 2011; Miyata and Kitagawa, 2017; Soma et al., 2017). However, the underlying cellular mechanisms governing laterality are unknown. Our novel finding that PNNs are expressed in a lateralized manner in mouse SS1 suggests that the lateralized expression could contribute to functional specialization. Pharmacological manipulations of removing PNNs unilaterally in SS1 subregions and exploring the functional consequences via electrophysiological and behavioral studies are ongoing. Previously we showed that, in this adult female mouse model for Rett syndrome (Het), PNNs were increased in a transient atypical manner in the auditory cortex, which correlated with their inefficient pup retrieval (Krishnan et al., 2017). Manipulating auditory cortex PNNs by ChABC injections or genetic reductions in Het significantly improved aspects of SH pup retrieval behavior, showing that PNNs play crucial roles in learning and executing this behavior (Krishnan et al., 2017). We recently showed that aberrant PNN expression leads to dysregulation of auditory cortical PV networks in Het, while behaviorally-relevant pup vocalization stimuli were presented during *in vivo* awake electrophysiological recordings (Lau et al., 2020). Here, we showed that NH already have abnormal increased PNN expression in subregions of SS1 compared with NW (Fig. 15, II), and surrogacy further increased PNN expressions (Fig. 15, III). Current results suggest information flow, network activation and multisensory integration could be affected in Het, in specific cortical regions such as SS1 and auditory cortex (Krishnan et al., 2017; Morello et al., 2018; Lau et al., 2020). Further whole brain analysis on laterality in PNN density in the auditory cortex is warranted, especially due to reports suggesting left hemisphere-specific neural circuitry activation (Ehret et al., 1987; Stiebler et al., 1997; Marlin et al., 2015).

### What regulates expression of PNN proteins?

Changes in PNN expression in the early developing cortex correlates with the increasing expression of MECP2, PV, and other components of the GABAergic machinery in PV+ GABAergic neurons (Krishnan et al., 2015). Expression of MECP2, PV (calcium binding protein), GAD67 (major enzyme that makes GABA in the cortex) and PNNs can dynamically change with activity, in both developing and adult brains (Ye and Miao, 2013; Krishnan et al., 2015, 2017). MECP2 directly occupies the promoter regions of *Gad1* (gene that makes GAD67) and *PV* (Chao et al., 2010; Durand et al., 2012), thus potentially configuring chromatin in these promoter and enhancer regions for appropriate activity-dependent and experience-dependent regulation. MECP2 regulates many genes (Cohen et al., 2011b; Gabel et al., 2015); therefore, genes encoding for PNN proteins could also be regulated by MECP2. Previously, we showed that the expression of mature PNNs is accelerated in the developing male *Mecp2*-null primary visual cortex (Krishnan et al., 2015), suggesting MECP2 could act as a repressor of PNNs. However, reducing GABAergic inhibition by using a *Gad1* Het allele in *Mecp2-*mutant backgrounds reduced PNN expression to WT levels (Krishnan et al., 2015, 2017), suggesting that expression of the extracellular matrix proteins that ultimately form PNNs may not require direct control of MECP2 and is amenable to change with dynamic inhibition changes.

### Mosaicism in MECP2 expression and *Het* variability

Most previous studies in mouse models of RTT were conducted in *Mecp2*-null male mice, because they exhibit earlier and more severe phenotypes in many standard assays. Therefore, with the exception of a few studies (Stearns et al., 2007; Garg et al., 2013; Samaco et al., 2013; Ribeiro and MacDonald, 2020), the molecular, circuit and behavioral defects in *Mecp2^Het^* female mice are largely unknown. Since RTT affects predominantly females, *Mecp2^Het^* female mice represent a more translationally relevant model of RTT than *Mecp2*-null male mice, though they are rarely used in studies due to random X chromosome inactivation patterns contributing to variability in phenotypes. In the alloparental behavioral model, we have shown that even with mosaic expression of MECP2 in the auditory cortex of Het, higher MECP2 expression correlates well with better efficiency during pup gathering behavior (Krishnan et al., 2017). MECP2 is known to have different levels in different cell types (Lioy et al., 2011; Lee et al., 2014; Song et al., 2014; Rakela et al., 2018). MECP2 protein function can be regulated by activity-dependent post-translational mechanisms (Cohen et al., 2011b; Ebert et al., 2013; Gabel et al., 2015); however, these studies do not take laterality or regional differences into account. Here, we find that MECP2 expression is asymmetric between left and right hemispheres of SS1, in both WT and Het with individual variations across brains (Figs. 11–14). These results suggest a more in-depth and systematic analysis is required to determine how variable MECP2 expression in hemispheres, subregions and cell types over time could result in diverse phenotypes. Maternal or paternal allele activation in individual cells should also be taken into consideration, as it could contribute to laterality in CNS cell types (Wu et al., 2014).

## Synthesis

Reviewing Editor: Christophe Bernard, INSERM & Institut de Neurosciences des Systèmes Decisions are customarily a result of the Reviewing Editor and the peer reviewers coming together and discussing their recommendations until a consensus is reached. When revisions are invited, a fact-based synthesis statement explaining their decision and outlining what is needed to prepare a revision will be listed below. The following reviewer(s) agreed to reveal their identity: Pietro Artoni.

This work by Lau et al. indicates that during the surrogate maternal experience there is a remapping of PNN in the somatosensory cortex. These results are novel and interesting, however the authors must clarify the following points, especially the main issues regarding choice of the mecp2-het mouse model.

Main issues:

1) As pointed out by the authors, mecp2 is a transcriptional regulator that controls plasticity. While it would make perfect sense to use mecp2 as a way to alter plasticity in the brain, a major concern is that, since mecp2 is a X-linked gene, the mecp2-het females used in the study are most likely strongly affected by mosaicism. Please refer to the following paper from Wu et al. on X inactivation in WT mice to consider how pronounced this effect may be (https://www.ncbi.nlm.nih.gov/pmc/articles/PMC3950970/). The manipulation of plasticity using the mecp2-het mutant may be a significant source of variability: because of the randomness of the first stages in X inactivation, there can be patches or large areas in the somatosensory cortex that are mainly mecp2+ or mainly mecp2-. So, when Naïve and Surrogated HET are compared, these two groups might have a completely different distribution of MecP2 KO cells and MecP2 WT cells. In which way is the mecp2 mosaicism responsible for altering these PNN maps? How is this issue mitigated or controlled in the study?

2) How do the extreme (but possible) X mosaicism cases (figure 6h in Wu et al., https://www.ncbi.nlm.nih.gov/pmc/articles/PMC3950970/), in which mecp2 would differentially be expressed across hemispheres, relate with the high abnormal variability in PNN concentration found mostly across hemispheres in naïve and less in naïve wild type littermates, as shown in figure 8?

Specific points Reviewer 1

1) Intro: 3rd paragraph mentions human brain and PNNs related to pathological conditions such as memory and motor agility. The authors should consider also mentioning the body of literature on other disease states such as schizophrenia and bipolar disorder.

2) Results: the authors need to consistently state what comparisons are being made. For example, in the abstract, ‘Mecp2-het females had increased PNN density in specific subregions in both hemispheres before maternal experience’... is this compared to WT? compared to naïve het? These should be stated throughout anytime statistical comparisons are being made.

3) A direct comparison of ‘naïve het’ to ‘naïve WT’ in the medial-> lateral graphs would be useful. The only data relevant to WT versus Het was presented in Table 1. It may not be possible with how the cohorts were run, in which case, the Table and the authors previous work should be cited.

4) PCA analysis: please define PC1 and PC2 in the figure legend or better yet, the figure to make it clear what variables are accounted for in PC1 vs PC2, eg. NH vs. SH (PC1).

5) Discussion: the discussion is short overall, and it could use more discussion on Mecp2 as a transcriptional repressor/ activator, and/or more on the possible mechanisms of regulating PNNs. There was one sentence on ‘MECP2 regulating tactile perception in SS1 (Orefice et al 2016)’ in the 2nd to last paragraph, but seems somewhat out of place/ confusing. This topic could also be expanded.

6) Throughout: Be careful with the wording, particularly use the of ‘after surrogacy, xxx increased’ (or similar phrases) since PNNs were not measured in individual animals before the experience (they were compared with naïve animals). Similarly, left is not increased over right (for example), it is higher/greater than the right (increased implies a change in response to treatment/behavior). I suggest staying with ‘surrogate animals had higher PPN staining/greater number of PNNs than naïve animals’... (as in other areas of the manuscript).

7) Be consistent with the format of the gene name when referring to the mice: MECP2 vs. MeCP2 vs. Mecp2. I believe it is italicized Mecp2, but please confirm nomenclature: http://www.informatics.jax.org/mgihome/nomen/gene.shtml

8) Color would be appropriate in the final figure, as this shade of grey was difficult to distinguish from black.

Specific points Reviewer 2

1) The stats in figure 4C’ do not seem to be supportive of the postulate (NW-L vs. SW-L). Significance is already shown in Table 1 (s), however, since this one of the major claims, consider significance for figure ’ as well. 

2) In the introduction and in the discussion, it is reported that PNN are “inhibitory to plasticity.” I suggest adopting a different adjective than "inhibitory." The term "inhibitory" may be misleading, as the reader may confuse the concept of closing plasticity via PNN maturation with the one of inhibitory activity (that is also strongly related to plasticity).

3) How is the threshold for the PNN detection established? The methods section reports as follows: "To count high-intensity mature PNNs, the ‘Contrast’ setting from the browser was set to the far right to threshold weaker signals." This sentence is not clear and requires clarification. I would recommend the authors to show the distribution of intensity of all PNNs and to indicate how the threshold is chosen based on this intensity distribution.

4) The phrase "we noticed a wide range distribution of PNN density across cohorts, ranging from 1 to 300" is unclear; the units are missing and should be added.

5) The order of citation of the panels in figure 2 flows oddly, starting from fig 2D, then going back to 2B, 2C, and never mentioning 2A. I suggest revising the order of these panels.

6) The scale in figure 3c is unclear. The numbers on the left are not aligned with ticks. Please clarify.

7) In figure 3, the lines indicating the average of the data seem to indicate a pattern from medial to lateral. This pattern appears consistently in all the panels 3A, 3B, 3C, and the variation in this pattern is comparable to the differences between NW vs. SW (3a), or SW-L vs. SW-R (3c). Hence the qestion: Is this pattern noise or is it statistically significant? The relevance of such a question lies in the fact that this pattern repeats systematically across hemispheres and mouse cohorts.

8) It seems that figure 3 is lacking statistical analysis, which is needed in order to support the following the statements made in the manuscript: "Changes in PNN density after surrogacy experience was seen in more medial sections. NW had similar PNN density across left (grey) and right (black) hemispheres (figure 3B), while SW showed hemisphere-specific changes in PNN density across the lateral-medial axis (Figure 3C)." Please include statistical analysis in your discussion of this section.

## Author Response

We thank the reviewers for the thoughtful questions and critiques. Overall, the reviewers found the results to be novel and interesting. We have addressed all the concerns here and in marked comments in the main manuscript. To address the main issue of mosaicism, we have added 4 new figures to the manuscript. We believe the manuscript is significantly improved. We address the critiques point-by-point below.

Manuscript Instructions

- Remove the Abbreviations paragraph/list.

This has been removed, and the abbreviations are defined where necessary.

Main issues:

1) As pointed out by the authors, mecp2 is a transcriptional regulator that controls plasticity. While it would make perfect sense to use mecp2 as a way to alter plasticity in the brain, a major concern is that, since mecp2 is a X-linked gene, the mecp2-het females used in the study are most likely strongly affected by mosaicism. Please refer to the following paper from Wu et al. on X inactivation in WT mice to consider how pronounced this effect may be (https://www.ncbi.nlm.nih.gov/pmc/articles/PMC3950970/). The manipulation of plasticity using the mecp2-het mutant may be a significant source of variability: because of the randomness of the first stages in X inactivation, there can be patches or large areas in the somatosensory cortex that are mainly mecp2+ or mainly mecp2-. So, when Naïve and Surrogated HET are compared, these two groups might have a completely different distribution of MecP2 KO cells and MecP2 WT cells. In which way is the mecp2 mosaicism responsible for altering these PNN maps? How is this issue mitigated or controlled in the study? 2) How do the extreme (but possible) X mosaicism cases (figure 6h in Wu et al., https://www.ncbi.nlm.nih.gov/pmc/articles/PMC3950970/), in which mecp2 would differentially be expressed across hemispheres, relate with the high abnormal variability in PNN concentration found mostly across hemispheres in naïve and less in naïve wild type littermates, as shown in figure 8?

We thank the reviewers for highlighting the mosaicism issue. This is a major problem in the field and one that we strive hard to take into consideration. In fact, in our original paper establishing alloparental behavior as an assay to study adult experience dependent plasticity (Krishnan, Lau et al, 2017), we specifically show how mosaic MECP2 expression in the adult auditory cortex correlates well with behavioral performance (Reviewer Figure 1). In our previous analysis in the auditory cortex and current analysis in the primary somatosensory cortex (new figure, 10-13), we predominantly see "salt and pepper" pattern of MECP2 expression. This result is in agreement with other papers (Metcalf BM et al, Neuroscience, 2006; Rietveld L et al, Frontiers in Cellular Neuroscience, 2015; and reviewed in Ribeiro M et al, Brain Research, 2020), especially in the age of our mice used in the study (7-12weeks old).

In Figure 11, MECP2 expression in individual left (A, B, C, D, E) and the corresponding right (A’, B’, C’, D’, E’) hemisphere across a cortical section containing S1BF of naïve Het female is imaged in a single plane. Individual variation across cohorts and hemispheres is observed. Histogram analysis of this expression shows that MECP2 expression can be significantly different between hemispheres, alternating between high left (B", D’, E’) and high right (A’). In Figure 11 A’, right hemisphere of cohort 1 shows a patch of missing MECP2 expression in the middle top area, by layers 1-3. However, this is a rare case (1/20). Furthermore, S1BF has predominant PNN expression in layer 4 and below (Figure 2, in manuscript), and not where this patch is found. As has been suggested in the field (and the above references), MECP2 can have cell autonomous and non-cell autonomous effects on neural circuitry structure and function.

In Figure 12, MECP2 expression in individual left (A, B, C, D, E) and the corresponding right (A’, B’, C’, D’, E’) hemisphere across a cortical section containing S1BF of SurHet female is imaged in a single plane. Individual variation across cohorts and hemispheres is observed. Histogram analysis of this expression shows that MECP2 expression can be significantly different between hemispheres, alternating between high left (B’, E’) and high right (A’, C’). Thus, we don’t think naïve and Sur Hets have completely different MECP2 distributions where one condition has more patchy expression over the other.

When we performed the same analysis on the WT (Figure 13, 14), we noticed similar left-right asymmetry bias in MECP2 expression, albeit at a higher level of expression (as WT have all cells expressing MECP2). Thus, we don’t think there is a direct connection between levels of MECP2 expression and PNN density.

MECP2, as a gene regulator can affect expression of ∼1000s of genes, as shown by the transcriptome studies from different labs. PNNs are formed by 40 different secreted proteins from different cell types. The extracellular matrix proteins are quite ubiquitous and can be detected in multiple cell types by transcriptomic analysis. However, they do not correlate well with protein expression studies, due to the transmembrane components embedded in the plasma membrane, which separate into insoluble compartments during protein extraction.

Furthermore, PNN expression is experience-dependent in WT as well, as shown in previous cited work. WT, in this case, expresses the correct level of MECP2 expression, unlike the Hets. Additionally, PNN expression is disrupted in other systems and models, such as in CDKL5 knockout mice, schizophrenia/bipolar disorder/Alzheimers’ animal models, where MECP2 expression is unlikely to be a leading cause. Thus, we think of PNNs as markers for experience dependent plasticity, as a proxy for PV+ GABAergic inhibitory networks. Here, we show they change in adult brains as well, under appropriate contexts. The direct or indirect mechanistic link is beyond the scope of this paper. Ideally, one would perform cell culture experiments to manipulate MECP2 expression with varying cell types to address this issue.

Regarding the wonderful Wu et al paper, we appreciate the reviewers bringing this paper to our attention. At present, in our studies, we do not have an approach to track if MECP2 is being expressed from the maternal or paternal allele in individual sections. We looked into obtaining the mice described in Wu et al and found that Jax has them cryopreserved. Hence, we are looking at alternatives currently. Suffice it to say that we see the individual variability in MECP2 and PNN expression in individual sections of WT as well, and not just in Hets. Thus, even if allelic expression could be a factor, it is unlikely to only affect Het phenotype.

We have incorporated this answer in the Discussion section of the manuscript as well.

Specific points Reviewer 1

1) Intro: 3rd paragraph mentions human brain and PNNs related to pathological conditions such as memory and motor agility. The authors should consider also mentioning the body of literature on other disease states such as schizophrenia and bipolar disorder.

We have now added three references that highlight PNN involvement in schizophrenia and bipolar disorder in this paragraph.

2) Results: the authors need to consistently state what comparisons are being made. For example, in the abstract, ‘Mecp2-het females had increased PNN density in specific subregions in both hemispheres before maternal experience’... is this compared to WT? compared to naïve het? These should be stated throughout anytime statistical comparisons are being made.

We thank the reviewer for this suggestion. We have incorporated these changes throughout the manuscript.

3) A direct comparison of ‘naïve het’ to ‘naïve WT’ in the medial-> lateral graphs would be useful. The only data relevant to WT versus Het was presented in Table 1. It may not be possible with how the cohorts were run, in which case, the Table and the authors previous work should be cited.

We thank the reviewer for this suggestion. We have included a new figure comparing ‘naïve WT’ with ‘naïve Het’ in Figure 7 (A-F), to accompany Table 1.

Lateral sections within each genotype have significantly higher density for PNNs, compared to medial sections within that genotype in whole brain and in a hemisphere-specific manner in both naive and Sur (Figure 7). Het display significantly higher density of PNNs compared to WT (Fig. 7, A, B for Naïve, D,E for Sur), in the more lateral sections and not in the medial sections, in the whole brain and left hemisphere. No significant differences in PNN density is observed in the right hemisphere, while comparing naïve WT to naïve Het (Figure 7, C) or Sur WT to Sur Het (F). Together these results add to the conclusion that lateral regions of the SS1 displayed increased PNN density, compared to medial sections; and that Het follow this overall pattern, however, with higher density than WT.

4) PCA analysis: please define PC1 and PC2 in the figure legend or better yet, the figure to make it clear what variables are accounted for in PC1 vs PC2, eg. NH vs. SH (PC1).

We thank the reviewer for this question. For panel A, we are plotting the projection of all individuals onto PC1 and PC2. These PCs are defined by relative weights of the different brain regions-all of the weights for PC1 and PC2 are shown in panel B.

While PC1 tends to distinguish surrogates from naïve (in particular, surrogate het from all others), the PCA itself is an unbiased analysis that is not designed to calculate the differences in "NH vs. SH". Rather, the PC1 represents the combination of factors that are the largest source of variation among all the samples. The PC1 itself is fully defined by the weights of the different brain sections. These weights (panel B) show a trend from medial to lateral, showing that differences in laterality are the largest source of variation in the data. This is analogous to the use of PCA in single cell RNA-seq to distinguish, for example, cell types in the brain. PC1 may tend to distinguish glia from GABAergic neurons, but the variables accounted for in PC1 would be a set of genes that have a high contribution to this PC. One could not say that PC1 is defined as "glia vs. GABAergic" even if this is what it tends to separate. We could only state that certain genes help distinguish glia from GABAergic neurons, the same way that in our analysis, certain brain regions help distinguish SH from other conditions.

5) Discussion: the discussion is short overall, and it could use more discussion on Mecp2 as a transcriptional repressor/ activator, and/or more on the possible mechanisms of regulating PNNs. There was one sentence on ‘MECP2 regulating tactile perception in SS1 (Orefice et al 2016)’ in the 2nd to last paragraph, but seems somewhat out of place/ confusing. This topic could also be expanded.

We have elaborated the Discussion section per reviewer’s guidance. We removed the reference to the Orefice paper, as we agree with the reviewer’s suggestion that it is out of place and confusing in this context.

6) Throughout: Be careful with the wording, particularly use the of ‘after surrogacy, xxx increased’ (or similar phrases) since PNNs were not measured in individual animals before the experience (they were compared with naïve animals). Similarly, left is not increased over right (for example), it is higher/greater than the right (increased implies a change in response to treatment/behavior). I suggest staying with ‘surrogate animals had higher PPN staining/greater number of PNNs than naïve animals’... (as in other areas of the manuscript).

We thank the reviewer for this suggestion. This issue has been corrected throughout the manuscript.

7) Be consistent with the format of the gene name when referring to the mice: MECP2 vs. MeCP2 vs. Mecp2. I believe it is italicized Mecp2, but please confirm nomenclature: http://www.informatics.jax.org/mgihome/nomen/gene.shtml

We have corrected the nomenclature throughout the manuscript to denote Mecp2 for gene and MECP2 for protein.

8) Color would be appropriate in the final figure, as this shade of grey was difficult to distinguish from black.

We have changed the "decreased" expression to light blue.

Specific points Reviewer 2:

1) The stats in figure 4C’ do not seem to be supportive of the postulate (NW-L vs. SW-L). Significance is already shown in Table 1 (s), however, since this one of the major claims, consider significance for figure 4C’ as well.

We agree with this point and have indicated significance in figure 4C’ now.

2) In the introduction and in the discussion, it is reported that PNN are "inhibitory to plasticity." I suggest adopting a different adjective than "inhibitory." The term "inhibitory" may be misleading, as the reader may confuse the concept of closing plasticity via PNN maturation with the one of inhibitory activity (that is also strongly related to plasticity).

We thank the reviewer for this question. However, we do think "inhibitory" is the right term to use here. As cortical PNNs are mainly found on parvalbumin+ fast spiking GABAergic interneurons and are thought to solidify inhibitory synapses onto each other, the net effect is better timing of inhibitory input onto postsynaptic neurons. Though we do not measure electrophysiological responses in this manuscript, we have previously shown increased PV+ interneuron inhibitory activity (and more inhibition) in the developing Mecp2-null visual cortex (Krishnan, Wang et al, PNAS 2015), and in adult Mecp2-heterozygous female auditory cortex (Lau BYB et al, Journal of Neuroscience, 2020).

3) How is the threshold for the PNN detection established? The methods section reports as follows: "To count high-intensity mature PNNs, the ‘Contrast’ setting from the browser was set to the far right to threshold weaker signals." This sentence is not clear and requires clarification. I would recommend the authors to show the distribution of intensity of all PNNs and to indicate how the threshold is chosen based on this intensity distribution.

We give an example here to clarify how the threshold was initially chosen, as we focus only on high-intensity PNNs for our analysis. These high-intensity PNNs are considered to be proxies for "mature" PV networks. These intensities and histogram plots change over subregions of the brain. We have now made detailed protocols for the mapping and counting procedures for high-intensity PNNs, with text and video. This is now available through protocols.io. dx.doi.org/10.17504/protocols.io.bcf8itrw

4) The phrase "we noticed a wide range distribution of PNN density across cohorts, ranging from 1 to 300" is unclear; the units are missing and should be added.

This sentence has been corrected. The main point was to emphasize the variability in PNN counts per section, even within individual brains.

5) The order of citation of the panels in figure 2 flows oddly, starting from fig 2D, then going back to 2B, 2C, and never mentioning 2A. I suggest revising the order of these panels.

We have added a description of the representative figures for 2A-C, before describing the quantification for 2D, to have better flow in text.

6) The scale in figure 3c is unclear. The numbers on the left are not aligned with ticks. Please clarify.

We have corrected this issue in the current version.

7) In figure 3, the lines indicating the average of the data seem to indicate a pattern from medial to lateral. This pattern appears consistently in all the panels 3A, 3B, 3C, and the variation in this pattern is comparable to the differences between NW vs. SW (3a), or SW-L vs. SW-R (3c). Hence the question: Is this pattern noise or is it statistically significant? The relevance of such a question lies in the fact that this pattern repeats systematically across hemispheres and mouse cohorts.

Due to the variability in the PNN density and sample size in each map position (the X axis ticks), we are not able to assess statistical significance between conditions (NW vs. SW). We agree with the reviewer that the average line indicates lateral sections have higher PNN density over medial sections. Hence, we combined PNN density in the two most lateral and medial sections and assessed statistical significance at the extremes. As marked in figure 3, we found lateral sections to have statistically significant PNN density over the medial sections in all the conditions ( NW vs. SW (A), NW-L vs. NW-R (B), SW-L vs. SW-R (C)), highlighting the regional distribution differences in PNN density. We have also performed similar analysis in figures 6 and 7.

8) It seems that figure 3 is lacking statistical analysis, which is needed in order to support the following the statements made in the manuscript: "Changes in PNN density after surrogacy experience was seen in more medial sections. NW had similar PNN density across left (grey) and right (black) hemispheres (figure 3B), while SW showed hemisphere-specific changes in PNN density across the lateral-medial axis (Figure 3C)." Please include statistical analysis in your discussion of this section.

We have now included statistical analysis, as elaborated in the above question.
